# Evidence of steady-state fibroblast subtypes in the normal human breast as cells-of-origin for perturbed-state fibroblasts in breast cancer

**DOI:** 10.1186/s13058-024-01763-3

**Published:** 2024-01-16

**Authors:** Mikkel Morsing Bagger, Jonas Sjölund, Jiyoung Kim, Katharina Theresa Kohler, René Villadsen, Abbas Jafari, Moustapha Kassem, Kristian Pietras, Lone Rønnov-Jessen, Ole William Petersen

**Affiliations:** 1 Division of Translational Cancer Research, Department of Laboratory Medicine, Lund University Cancer Centre, Lund University, Lund, Sweden; 2 Department of Cellular and Molecular Medicine, University of Copenhagen, Copenhagen, Denmark; 3 Laboratory of Molecular Endocrinology, KMEB, Department of Endocrinology, Odense University Hospital and University of Southern Denmark, Odense, Denmark; 4 Section for Cell Biology and Physiology, Department of Biology, University of Copenhagen, Copenhagen, Denmark

**Keywords:** Breast cancer, Cancer-associated fibroblast, Fibroblast, iCAF, myCAF, Cell line, TDLU

## Abstract

**Background:**

Human breast cancer most frequently originates within a well-defined anatomical structure referred to as the terminal duct lobular unit (TDLU). This structure is endowed with its very own lobular fibroblasts representing one out of two steady-state fibroblast subtypes—the other being interlobular fibroblasts. While cancer-associated fibroblasts (CAFs) are increasingly appreciated as covering a spectrum of perturbed states, we lack a coherent understanding of their relationship—if any—with the steady-state fibroblast subtypes. To address this, we here established two autologous CAF lines representing inflammatory CAFs (iCAFs) and myofibroblast CAFs (myCAFs) and compared them with already established interlobular- and lobular fibroblasts with respect to their origin and impact on tumor formation.

**Methods:**

Primary breast tumor-derived CAFs were transduced to express human telomerase reverse transcriptase (hTERT) and sorted into CD105^low^ and CD105^high^ populations using fluorescence-activated cell sorting (FACS). The two populations were tested for differentiation similarities to iCAF and myCAF states through transcriptome-wide RNA-Sequencing (RNA-Seq) including comparison to an available iCAF-myCAF cell state atlas. Inference of origin in interlobular and lobular fibroblasts relied on RNA-Seq profiles, immunocytochemistry and growth characteristics. Osteogenic differentiation and bone formation assays in culture and in vivo were employed to gauge for origin in bone marrow-derived mesenchymal stem cells (bMSCs). Functional characteristics were assessed with respect to contractility in culture and interaction with tumor cells in mouse xenografts. The cells’ gene expression signatures were tested for association with clinical outcome of breast cancer patients using survival data from The Cancer Genome Atlas database.

**Results:**

We demonstrate that iCAFs have properties in common with interlobular fibroblasts while myCAFs and lobular fibroblasts are related. None of the CAFs qualify as bMSCs as revealed by lack of critical performance in bone formation assays. Functionally, myCAFs and lobular fibroblasts are almost equally tumor promoting as opposed to iCAFs and interlobular fibroblasts. A myCAF gene signature is found to associate with poor breast cancer-specific survival.

**Conclusions:**

We propose that iCAFs and myCAFs originate in interlobular and lobular fibroblasts, respectively, and more importantly, that the tumor-promoting properties of lobular fibroblasts render the TDLU an epicenter for breast cancer evolution.

**Supplementary Information:**

The online version contains supplementary material available at 10.1186/s13058-024-01763-3.

## Background

Recent advances in both single-plex and high-dimensional analyses have expanded the repertoire of normal fibroblast subtypes and CAFs attributable to tissue homeostasis and tumor modulation, respectively [1, 2]. In the human breast, the potential source of fibroblasts has been resolved to the extent of anatomical locations in either loosely arranged TDLU stroma or more densely packed interlobular stroma (reviewed in [3]). The TDLU is the most dynamic element of the breast during puberty, menstrual cycle, pregnancy, and involution. By comparison, the interlobular ducts are less dynamic. The anatomical organization of the stroma is orchestrated by separate fibroblast lineages characterized by individual phenotypic profiles. TDLU-associated lobular fibroblasts are CD105^high^/CD26^low^, bMSC-like and facilitate branching morphogenesis of parenchymal epithelial cells in a transforming growth factor-beta (TGF-β)-dependent manner [4, 5]. Interlobular fibroblasts, on the other hand, are CD105^low^/CD26^high^ and direct the ductal epithelial differentiation repertoire in culture and in vivo [5].

In breast cancer, the stroma is distorted to a degree where it is no longer possible to discern the structural differences between lobular and interlobular, and thus, it remains a major challenge to make connections between CAFs and resident fibroblasts. This makes it difficult to assess exactly the nature of conversions that the CAFs have undergone as a consequence of the fibroblasts’ exposure to the tumor microenvironment. We have previously identified tumor-derived TGF-β as the major cytokine responsible for conversion of resident fibroblasts into CAFs [6] and moreover revealed significant diversity in cellular composition of tumor stroma [7]. Indeed, the cell of origin of human breast CAFs has been shown to include resident fibroblasts, perivascular fibroblasts, blood vessel mural cells, and the malignant clone itself ([6–10] and reviewed in [3]). Among these potential sources, the resident fibroblasts are by far the most frequent ([7] and reviewed in [3]). However, recent single-cell sequencing efforts have revealed a spatial and temporal heterogeneity among breast CAFs, which is difficult to explain from the point of view of a single fibroblastic origin [10–17].

Here, we establish CAF cell lines from three human estrogen receptor-positive (ER^+^) breast carcinomas and investigate their resemblance with iCAFs and myCAFs and with normal-derived interlobular- and lobular fibroblasts, respectively. We demonstrate that upon transplantation to mice together with ER^+^ human breast cancer cells, only normal-derived lobular fibroblasts and myCAFs increase tumor volume over that of cancer cells transplanted alone. The apparent tumor supportive properties are further confirmed by association of the myCAF signature with poor breast cancer survival.

## Results

### Maintenance of myCAF and iCAF properties in established cell lines

**Fig. 1:**
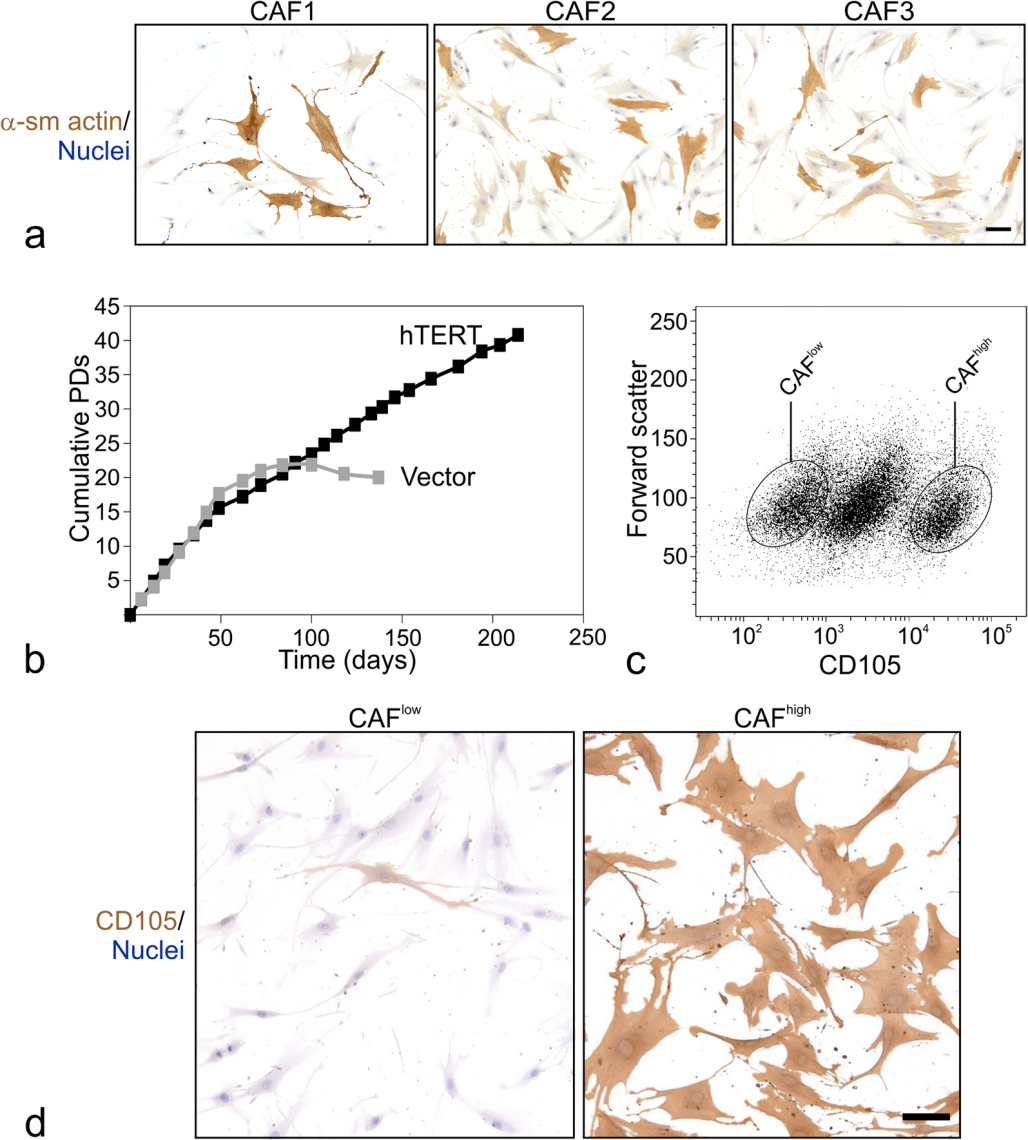
Isolation and immortalization of stably propagated CD105^low^ and CD105^high^ CAF states. **a** Micrographs of hTERT-transduced crude CAF cultures from three primary breast cancer biopsies cultured to passage seven and immunoperoxidase-stained for α-sm actin (brown) and counterstained by hematoxylin for nuclei (blue). Scale bar = 50 μm. **b** Diagram of growth characteristics depicted as the cumulative population doublings (PDs) versus time (days) in CAF1 transduced with hTERT or empty vector. Note the extended life span of the hTERT-transduced CAF1 cells reaching > 40 PDs, whereas the empty vector-transduced cells refrain from doubling after ~ 20 PDs. **c** FACS dot plot of single-cell suspended CAF1 in passage eight labeled with a CD105 antibody and plotted versus forward scatter. Circles indicate the approximate CD105^low^ (CAF^low^) and CD105^high^ (CAF^high^) populations isolated for further subculture. **d** Micrographs of CAF1-derived CAF^low^ and CAF^high^ stained by immunoperoxidase against CD105 (brown) and with hematoxylin for nuclei (blue). Note the relative intense CD105 staining in CAF^high^ relative to CAF^low^. Scale bar = 50 μm

iCAFs and myCAFs are among the most frequent, non-neoplastic cell types in human breast solid tumors, and numerous predictions have been made about their function based on molecular profiling [13, 14, 17]. However, experimental evidence for these predictions is still lagging behind due to insufficient access to specific cell states within the fibroblast-to-CAF spectrum. To address this issue, we isolated and immortalized CAFs from primary breast carcinomas. The presence of α-smooth muscle (sm) actin-positive CAFs in all tumor-derived cultures was demonstrated by immunocytochemical staining of primary cultures from eleven randomly chosen biopsies (data not shown) and long term cultures from three additional biopsies (CAF1, CAF2, CAF3) transduced to express hTERT (Fig. 1a). CAF1, CAF2, and CAF3 were randomly selected from a series of consecutively collected breast cancer biopsies, which by immunohistochemical staining for GATA3, ER, progesterone receptor (PR), cytokeratin 5 (K5), cytokeratin 14 (K14) and human epidermal growth factor receptor 2 (HER2) were classified as ER^+^ luminal breast carcinomas (Table 1). Hence, the three CAF lines derive from the most frequent breast cancer subtype. One of the long-term cultures, CAF1, was followed in extended culture. After a brief period of stagnation, hTERT-transduced CAF1 grew exponentially without signs of senescence for 40 + population doublings (Fig. 1b). Control cells with empty vector grew similar to primary cells without transduction until senescence after approximately two months corresponding to twenty population doublings (Fig. 1b). We originally showed that two fibroblast subtypes can be distinguished in human normal breast by their distinct expression patterns of CD105 (Endoglin, an auxiliary receptor within the TGF-β signaling complex) and CD26 (dipeptidyl-peptidase IV), and that CD105 is the most stable marker upon extended culture [4, 5]. Here, we therefore enriched for populations among CAF1 with potentially differing iCAF versus myCAF traits by FACS using CD105. Our FACS protocol revealed CD105^low^ (hereafter referred to as CAF^low^) and CD105^high^ (hereafter referred to as CAF^high^) populations (Fig. 1c). Similar populations, albeit at different frequencies, could be isolated from extended cultures of hTERT immortalized fibroblasts of the two other primary carcinomas, CAF2 and CAF3 (Additional file 1: Fig. S1a). An accumulation of α-sm actin-positive CAFs in the CAF^high^ gate was seen in both CAF1 and CAF2 (Additional file 1: Fig. S1b). Indeed, a mixture of CAF^low^ and CAF^high^ was found also in situ in the tumor of origin of CAF1, CAF2 and CAF3 (Additional file 2: Fig. S1c). The presence of CAF^low^ and CAF^high^ in breast carcinomas was further confirmed in nineteen out of a small sample of twenty-seven primary carcinomas. Both CAF1-derived CAF^low^ and CAF^high^ were readily propagated under identical culture conditions, and they have currently undergone a minimum of 38 population doublings. A seemingly intermediate population between CAF^low^ and CAF^high^ in CAF1 (Fig. 1c) was not pursued in the present study. Importantly, when examined by immunocytochemistry, CD105 levels remained low and high in the two populations, respectively, as determined up to passage 30 implicating stable phenotypes after isolation (Fig. 1d).

**Table 1:** CAF1, CAF2 and CAF3 derive from ER^+^ luminal breast cancers

Tumor	GATA3	ER	PR	Keratin 5	Keratin 14	HER2
CAF1	+	+	−	−	−	Low
CAF2	+	+	+	−	−	Low
CAF3	+	+	−	−	−	Low

Breast cancer subtype determination was performed by peroxidase immunohistochemistry on cryosectioned tissue. Tumors were considered positive (+) or negative (−) if more or less than 1% of the neoplastic cells were reactive with an antibody, respectively. Estrogen receptor (ER) was positive in more than 50% of neoplastic cells in CAF1, CAF2 and CAF3 tumors. Intensity of HER2 stainings was evaluated against control tissue with known high and low expression levels of HER2. GATA3: Gata binding protein 3. PR: Progesterone receptor. HER2: Human epidermal growth factor receptor 2

**Fig. 2:**
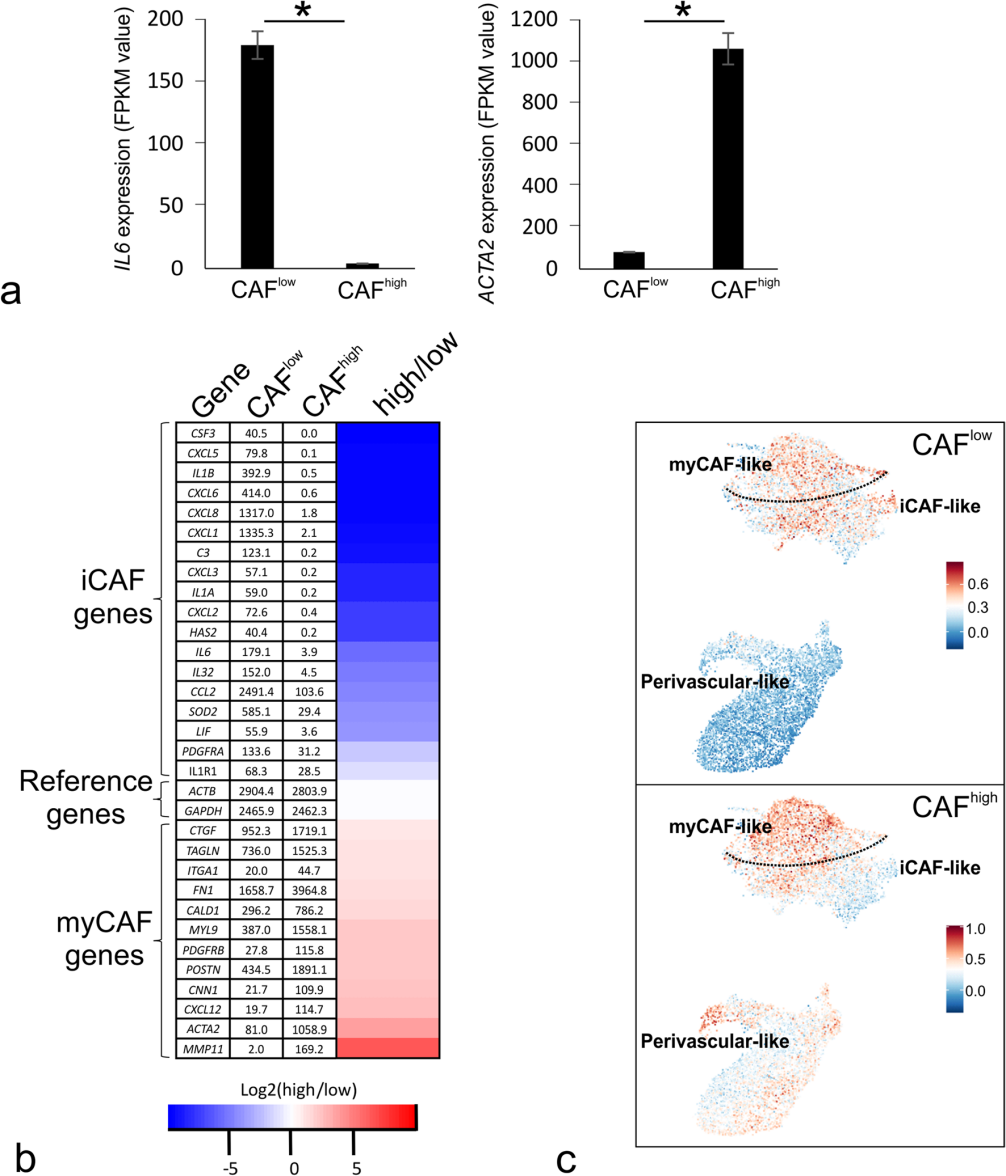

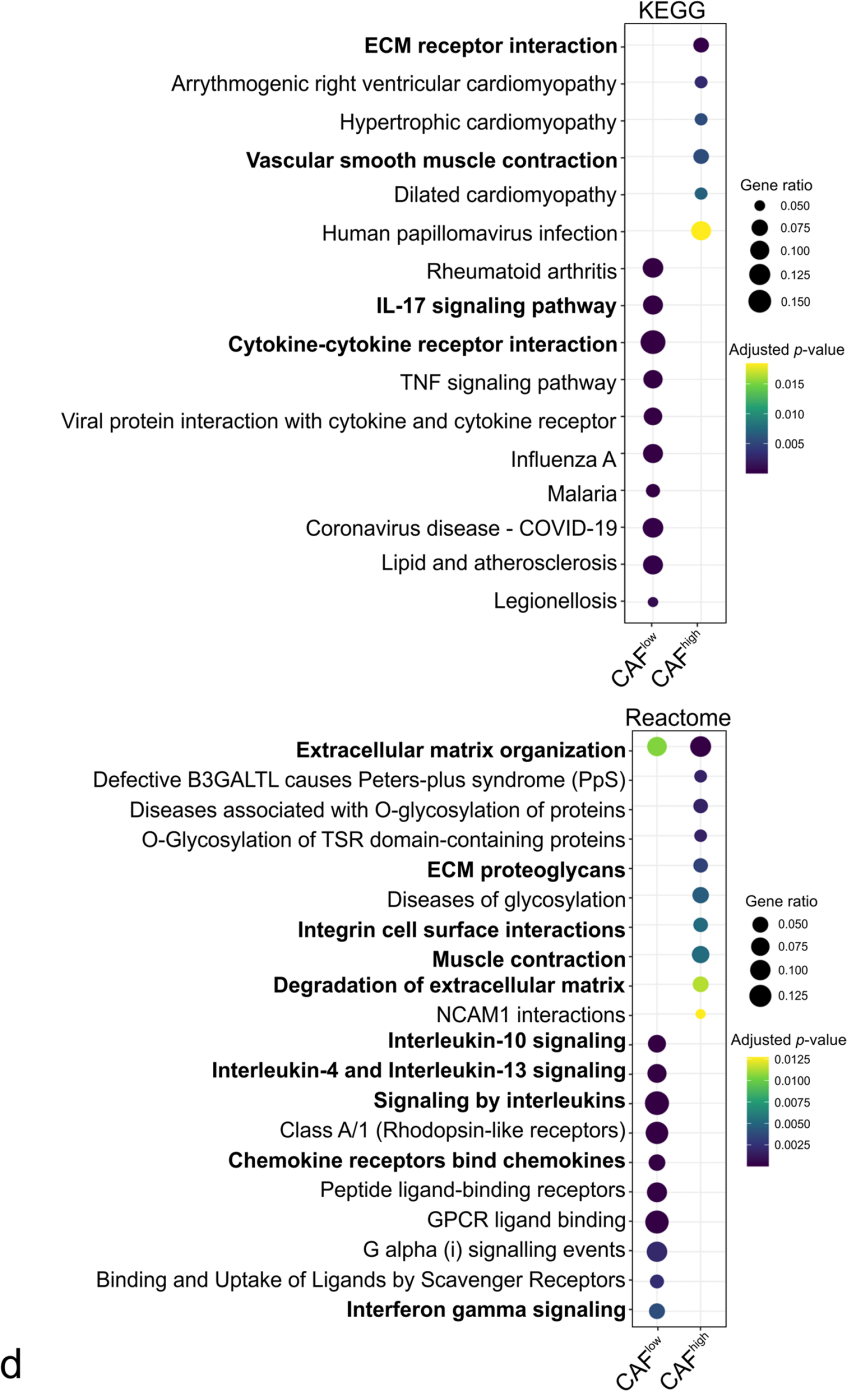
CAF^low^ resemble iCAFs and CAF^high^ resemble myCAFs. **a** Bar plots showing mean of transcript levels of the iCAF marker *IL6* and myCAF marker *ACTA2* in CAF^low^ and CAF^high^. Asterisks (*) indicate adjusted *p* < 0.001. Error bars represent ± standard deviation (SD). **b** Heatmap representation and FPKM levels of differentially expressed iCAF and myCAF genes (≥ 5 FPKM, ≥ 1.5-fold change, adjusted *p* < 0.001) by RNA-Seq in CAF^low^ and CAF^high^. *GAPDH* and *ACTB* are included as reference genes. Color key represents the logarithm (base 2) of the CAF^high^/CAF^low^ ratio. **c** Signature scores for each CAF^low^ (upper) and CAF^high^ (lower) cell line visualized onto Unifold Manifold Approximation and Projection (UMAP) representations of stromal cell types reproduced from a published breast cancer single-cell RNA-Seq dataset [17]. The color keys indicate the signature scores. iCAF-like and myCAF-like in the UMAPs refer to the nomenclature used in [17], and the dotted line has been applied to visualize the approximate division of these two clusters. While the CAF^low^ has a representation within the iCAF-like and myCAF-like cluster, the CAF^high^ is represented primarily in the myCAF-like cluster. **d** Dot plots showing KEGG (upper) and Reactome (lower) pathways significantly enriched when comparing CAF^low^ and CAF^high^ signatures. Size key indicates the ratio of enriched genes to background genes (top key). Color key indicates the Benjamini–Hochberg-adjusted *p*-value (bottom key). Key terms in bold text

To establish a link between CAF^low^ and CAF^high^ on one hand and iCAFs and myCAFs on the other, RNA-Seq was performed on CAF1 from which we recovered data for *ACTA2* and *IL6*. Indeed, *IL6*, which is considered an unequivocal marker of iCAFs was forty-six fold increased in CAF^low^ while the myCAF marker *ACTA2* was approximately 13- fold higher in CAF^high^ [18] (Fig. 2a). *IL6* and *ACTA2* remain the prototypical markers for the iCAF/myCAF phenotypes, but the criteria for defining iCAFs have expanded to encompass cytokines such as *CSF3*, *CXCL5*, *IL1B*, *CXCL8*, *CXCL1*, *CXCL2*, *CCL2*, *SOD2* and non-cytokines *C3* and *PDGFRα* ([19–21], and reviewed in [22]). As depicted in Fig. 2b, CAF^low^ expressed these iCAF markers, whereas CAF^high^ exhibited increased expression of prototypical myofibroblast markers including *CTGF*, *TAGLN*, *CALD1*, *MYL9*, *POSTN*, *CNN1*, *CXCL12* and *MMP11* [19, 23, 24].

The recent surge in single-cell RNA sequencing has resolved the CAF spectrum into multiple clusters among which iCAFs and myCAFs are the most prominent also in breast cancer [13, 14, 17]. By mapping the genes abundantly and significantly differentially expressed (≥ 100 FPKM, ≥ 2-fold change, adjusted *p* < 0.0001) between CAF^low^ and CAF^high^ onto a well-annotated human breast CAF atlas [17], we found that CAF^high^ map primarily with myCAFs and, to some extent, perivascular-like (PVL) cells, which is in good agreement with the myofibroblastic phenotype to include origin from fibroblasts as well as perivascular stromal cells [7, 8] (Fig. 2c). In contrast, CAF^low^ had a larger representation within the iCAF cluster (Fig. 2c).

To further characterize CAF^low^ and CAF^high^, we performed pathway enrichment analysis including the Kyoto Encyclopedia of Genes and Genomes (KEGG) and Reactome pathway databases, which among others, indicated enrichment of the IL17 signaling pathway, cytokine–cytokine receptor interaction and signaling by interleukins in CAF^low^ and ECM–receptor interaction, vascular smooth muscle contraction and integrin cell surface interactions modules in CAF^high^ (Fig. 2d and Additional file 3: Table S1).

Six iCAF markers (*C3*, *CXCL1*, *CXCL6*, *CXCL8*, *IL1B*, and *PDGFRA)* and six myCAF markers (*ACTA2*, *CALD1*, *CNN1*, *FN1*, *ITGA1*, and *TAGLN)* that were identified by RNA-Seq were all validated to be coordinately and statistically significantly different between CAF^low^ and CAF^high^ derived from CAF1 by RT-qPCR (Additional file 4: Fig. S2a). Despite some variation, this pattern of expression was essentially reproduced in CAF^low^ and CAF^high^ derived from CAF2 and CAF3 (Additional file 4: Fig. S2a). Moreover, and in agreement with the RNA expression patterns, protein staining for α-sm actin revealed that CAF^high^ from all three origins was enriched for myofibroblasts (Additional file 4: Fig. S2b). Notably, CAF1-derived CAF^high^ exhibited positive staining for α-sm actin in ~ 99% of the cells, and this phenotype was stably propagated from isolation in passage eight to its last recording in passage 39 (data not shown).

Collectively, our data are in strong favor of the CAF^low^ and CAF^high^ cell lines representing iCAFs and myCAFs of primary breast cancer. Based on these findings, CAF1-derived CAF^low^ and CAF^high^ are hereafter referred to as iCAFs and myCAFs, respectively.

### Lineage relationship between CAFs and fibroblasts

**Fig. 3:**
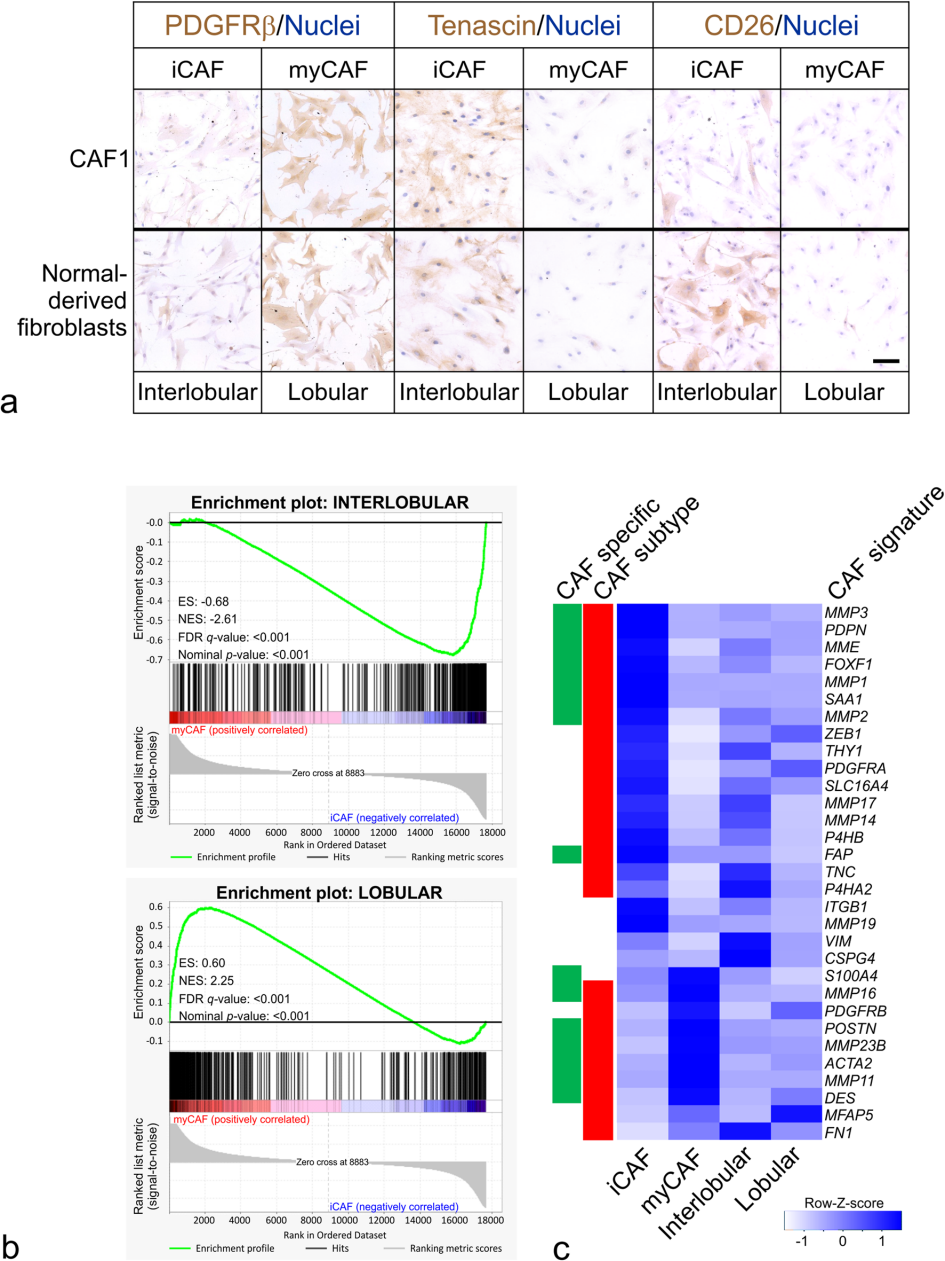
iCAFs and myCAFs reside in the interlobular and lobular steady states, respectively. **a** Micrographs of immunoperoxidase-stained cultures from CAF1 and normal-derived fibroblasts showing coordinated expression of PDGFRβ and tenascin within interlobular fibroblasts and iCAFs as well as within lobular fibroblasts and myCAFs independently of state. By comparison, the interlobular lineage marker, CD26, is state-dependent. Nuclei counterstained by hematoxylin. Scale bar = 50 μm. **b** Gene set enrichment analysis (GSEA) plots depicting significant enrichment between iCAFs and an interlobular gene set (upper) and a significant enrichment between myCAFs and a lobular gene set (lower). ES: Enrichment Score. NES: Normalized Enrichment Score. FDR: false discovery rate. **c** A heatmap representing a CAF signature of 31 literature curated CAF markers in iCAFs, myCAFs, interlobular and lobular fibroblasts. The red bar indicates the 26 CAF subtype-specific markers differentially expressed between iCAFs and myCAFs (≥ 5 FPKM, ≥ 2-fold change, adjusted *p* < 0.01). The green bar indicates the 15 CAF-specific markers elevated in either CAF type compared to both steady-state fibroblasts (≥ 5 FPKM, ≥ 2-fold change, adjusted *p* < 0.01). Note that most CAF markers exhibit CAF subtype-specific expression and that none of the CAF markers are elevated in the normal-derived interlobular or lobular fibroblasts compared to both perturbed states. The CAF signature is based on [26, 28, 29]. Key indicates row-*z*-score

The above observations led us to speculate on whether our previously described findings of two steady-state fibroblast subtypes in the normal human breast have any lineage relationship with the present perturbed-state CAF subtypes. To assess this, we applied our normal breast-derived CD105^low^/CD26^high^ interlobular and CD105^high^/CD26^low^ lobular fibroblast cell lines [5] and compared them with the CAF cell lines in terms of staining for the commonly applied markers platelet-derived growth factor-beta (PDGFRβ, tenascin and CD26 ([4, 25] and reviewed in [26]) (Fig. 3a). In general, we found a striking resemblance between interlobular fibroblasts and iCAFs as well as between lobular fibroblasts and myCAFs (Fig. 3a). However, in agreement with a reduction during CAF conversion [27], we noted a relatively low CD26 staining in iCAFs compared to interlobular fibroblasts (Fig. 3a). Similar staining profiles were observed in CAF2- and CAF3-derived CAF^low^ and CAF^high^, albeit the CAF^high^ lobular-like profile was less prominent in CAF3 (Additional file 5: Fig. S3a). To explore the relationship between normal-derived fibroblasts and CAFs further at the transcriptomic level, we utilized the RNA-Seq dataset and compared this with a previously generated RNA-Seq dataset from normal-derived fibroblasts [5] with respect to differentially expressed genes (≥ 2-fold change and ≥ 5 FPKM, adjusted *p* < 0.05) between interlobular- and lobular fibroblasts. Indeed, iCAFs overlapped significantly with an interlobular fibroblast gene set, whereas myCAFs exhibited significant enrichment in a lobular fibroblast gene set (Fig. 3b). This pattern of expression was validated by RT-qPCR in the normal-derived fibroblasts, iCAFs and myCAFs, and a similar pattern was observed in CAF2 and to some extent also in CAF3 (Additional file 5: Fig. S3b).

To further investigate the differentiation of the two CAF subtypes, we constructed a CAF signature of traditional CAF markers from contemporary literature reviews [26, 28, 29] and applied this to the CAF RNA-Seq dataset and the normal-derived fibroblast RNA-Seq dataset [5] (Fig. 3c). However, rather than being equally expressed, which might be expected from traditional CAF markers, an impressive 26 out of 31 were instead differentially expressed between iCAFs and myCAFs, including *MMP3*, *FAP* and *PDPN*, which were elevated in iCAFs and *POSTN*, and *FN1* which were elevated in myCAFs (Fig. 3c). Next, a potential resemblance of CAFs with bMSCs was addressed. However, both crudeCAFs and myCAFs failed to differentiate like bMSCs in both an osteogenic induction medium and a hydroxyapatite in vivo assay, which precludes a bone marrow origin of CAFs ([30–32] reviewed in [33]) (Additional file 6: Fig. S4a, Additional file 7: Fig. S4b, c). Also, all cells stained negative for endothelial CD31 and epithelial K19, and less than five percent of the cells were positive for perivascular markers MCAM and MYH11 (data not shown), thus further supporting a fibroblastic origin.

**Fig. 4:**
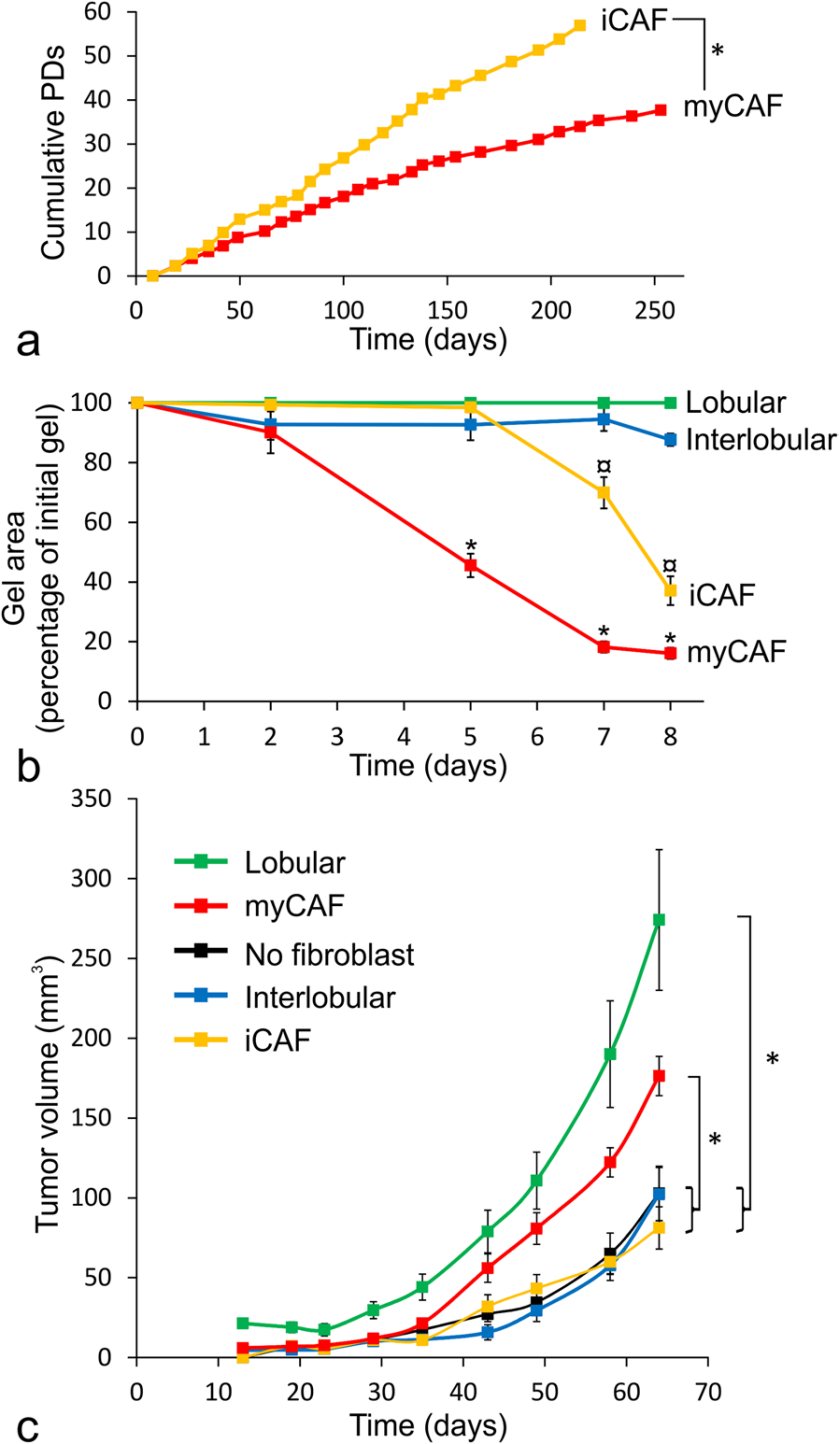
Fibroblast functional characteristics are inherent to lineage or are acquired state-dependent. **a** Diagram of growth characteristics depicted as the cumulative population doublings (PDs) versus time (days) of iCAFs (yellow) and myCAFs (red) from their isolation in passage eight to passage 31 and 34, respectively. iCAFs grew on average 2.47 PDs ± 0.54 SD per passage and myCAFs grew on average 1.40 PDs ± 0.30 SD per passage (asterisk indicates *p* < 0.001, unpaired Student’s *t*-test). The significant growth advantage of iCAFs over myCAFs is reminiscent of the growth advantage of interlobular over lobular fibroblasts [4, 5]. **b** Line graph of quantified gel area expressed in percentage of initial gel in a contraction assay determined on day 2, 5, 7 and 8 for iCAFs (yellow), myCAFs (red), interlobular fibroblasts (blue) and lobular fibroblasts (green). * above myCAFs indicate significantly smaller gels versus any other group on the same day (*p* < 0.05). ¤ above iCAFs indicate significantly smaller gels versus interlobular- and lobular fibroblasts on the same day (*p* < 0.05). Statistical test used was one-way analysis of variance (ANOVA) with Benjamini–Hochberg multiple test correction. Error bars represent ± standard error of the mean (SEM). **c** Growth curve of mean tumor volume (mm^3^) in mice injected with MCF7 breast cancer cells without fibroblasts (black) or with iCAFs (yellow), myCAFs (red), interlobular fibroblasts (blue) or lobular fibroblasts (green). Tumors with lobular fibroblasts and myCAFs were significantly larger than with interlobular fibroblasts, iCAFs and without fibroblasts on days 43, 49, 58 and 64 (endpoint) as indicated by curly brackets and asterisks (*, *p* < 0.05 by one-way ANOVA with Benjamini–Hochberg multiple test correction). Error bars represent ± SEM

Some general features distinguished CAFs from steady-state-like fibroblasts. Thus, in the CAF signature (Fig. 3c), 15 out of the 31 CAF markers were CAF-specific as they were minimum twofold higher in either of the CAFs compared to the interlobular- or lobular steady-state fibroblasts, which was a statistically significant enrichment (*p* < 0.001, Fisher’s exact test). In contrast, as expected, none of the CAF markers were higher in either of the steady-state fibroblasts compared to any of the CAFs (Fig. 3c). Although iCAFs and myCAFs differed from each other by growth rate (Fig. 4a) as do normal-derived fibroblasts [4, 5], both contracted collagen gels (Fig. 4b), which is a functional feature of CAFs [23]. Collectively, our data are in favor of the two major human breast steady-state fibroblast subtypes, interlobular and lobular, serving as precursors for the major perturbed-state CAF subtypes, i. e. iCAFs and myCAFs, respectively.

**Perturbed-state- and steady-state fibroblast subtypes are functionally related**

Despite the rising appreciation of transcriptionally defined iCAFs and myCAFs in the spectrum of perturbed fibroblast states, it remains unresolved if this manifests itself in differential support of tumor growth. In a first attempt to address this, we took advantage of now having established autologous CAF cell lines from *bona fide* primary ER^+^ breast cancer and used these to establish xenografts by co-implantation with ER^+^ MCF7 breast cancer cells. Rather than the two CAFs being uniformly supportive of tumor growth, as opposed to the steady-state fibroblasts as one might expect, the property of promoting growth is shared by the myCAFs and the lobular subtypes of perturbed- and steady-state fibroblasts, respectively (Fig. 4c).

**Fig. 5:**
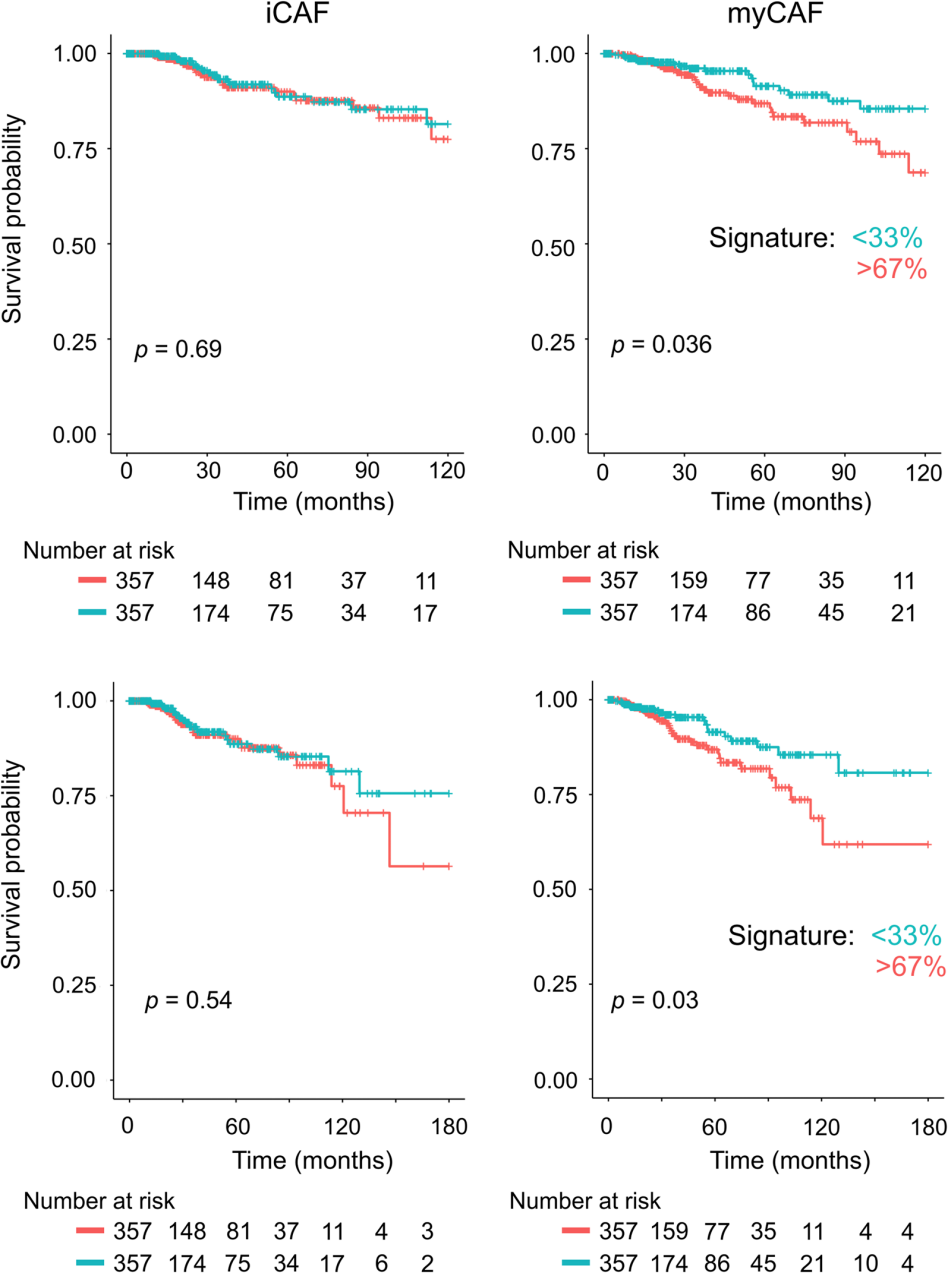
A myCAF gene signature associates with poor breast cancer survival**.** Kaplan–Meier curve showing significant association between the patients with a high (> 67%, red) myCAF gene signature (≥ 100 FPKM, ≥ 2-fold change, adjusted *p* < 0.0001) and poor breast cancer-specific survival as compared to the patients with a low (< 33%, blue) signature expression with 10-year follow-up (upper, *p* = 0.036) and 15-year follow-up (lower, *p* = 0.03) in data from TCGA. No significant association was found for patients with a high (> 67%, red) versus low (< 33%, blue) iCAF gene signature. Association was tested using the log-rank test. Number of patients at risk at the indicated time points are shown below each plot

We then addressed if such tumor supportive property among myCAFs could also be deduced from patient data from The Cancer Genome Atlas (TCGA) database using gene expression signatures of genes highly expressed (≥ 100 FPKM) and differentially expressed (≥ 2-Fold change, adjusted *p* < 0.0001) between iCAFs and myCAFs. While we did not observe any association of the iCAF signature with breast cancer-specific survival (Fig. 5), the myCAF signature exhibited significant association with poor breast cancer survival at 10-year and 15-year follow-up (Fig. 5). The myCAF signature was, however, not significantly associated with survival in a particular breast cancer molecular (PAM50 classification) subtype (Additional file 8: Fig. S5).

Collectively, these data are in favor of the subtype relationship between CAFs and normal-derived fibroblasts overriding perturbation-related changes in facilitating tumor evolution. The findings further underscore the significance of the stroma as key in the TDLUs’ susceptibility to tumor initiation.

## Discussion

CAFs have been implicated in regulating tumor cells directly through paracrine interactions and indirectly through regulation of almost all cellular as well as non-cellular constituents of the tumor microenvironment. Such multifaceted functionality has been increasingly difficult to reconcile with a single cell-of-origin (reviewed in [34]). With single-cell RNA-Seq, CAFs are now transcriptionally resolved largely into iCAFs and myCAFs, each suggested to exert specific cellular functions [13, 15]. However, characterization of iCAFs and myCAFs above the level of transcriptomics has lagged behind not least owing to the scarcity of cellular models available. Arguably, the most prominent source of CAFs is the steady-state fibroblast ([7] and reviewed in [3]). In our previous work, we delineated two steady-state fibroblasts in normal breast stroma: one in the TDLU, characterized by high CD105 and low CD26 expression, and another with comparatively low CD105 and high CD26 expression residing in the interlobular stroma [4, 5]. Already at the steady-state level, the lobular lineage exhibits a myofibroblastic gene expression profile versus the interlobular lineage, which instead expresses immune-related genes [4]. This observation prompted us to hypothesize that in the course of tumor evolution, the steady-state interlobular and lobular lineages are perturbed to the iCAF and myCAF states, respectively. In the present work, we demonstrate that CAF^low^ and CAF^high^ co-exist in situ and that these profiles can be recovered in cultured CAFs by FACS from three biopsies. We found that the relative proportions of CAF^low^ and CAF^high^ differed quite extensively between CAF1, CAF2 and CAF3 in culture. While this may reflect a heterogeneity between the tumors of origin with respect to CAF^low^ and CAF^high^ abundance, it cannot be ruled out that it relates to experimental or technical variation associated with, for example, cell selection or the hTERT transduction protocol. Efforts to unequivocally quantify the abundance of each type of fibroblast in situ are ongoing in our laboratories. Importantly, however, upon a single cycle of cell sorting, CD105 levels remained low and high, respectively, indicating that, not unlike normal breast fibroblasts [4, 5], the phenotypes of the sorted cells could be readily propagated also in long term. We further pursued the prospectively sorted cells from CAF1 and show through pathway analysis, gene expression profiling and immunostainings, that while CAF^low^ resemble iCAFs, CAF^high^ resemble myCAFs. Similar iCAF and myCAF profiles were revealed by RT-qPCR and immunostaining in CAF^low^ and CAF^high^, respectively, isolated from two additional biopsies, CAF2 and CAF3. In the latter, however, the iCAF and myCAF profiles appeared relatively weak. Future experiments should validate further the pathways enriched in the iCAFs and myCAFs. Nevertheless, our observation that iCAFs and myCAFs could be recovered albeit with some variation in three consecutively collected tumor biopsies, suggests that CD105 is a useful marker to enrich for those states by FACS, at least when it comes to luminal ER^+^ breast cancer. That it might also apply to triple-negative and HER2 breast cancer seems likely, considering that the single-cell atlas used for mapping of the cell lines with iCAF and myCAF clusters was generated as a composite of luminal ER^+^, HER2 and triple-negative breast cancers [17], but this awaits further scrutiny. It is, nevertheless, tempting to speculate that CD105 might be of use as a universal marker to enrich for iCAFs and myCAFs, since it has also been reported as a marker of pancreatic myCAFs [35].

One unanswered question from the RNA-based characterization of CAFs pertains to whether or not the iCAF/myCAF clusters should be taken to represent reversibly or irreversibly perturbed states (reviewed in [34]). Here, we recorded the iCAF/myCAF states in cells having undergone more than 35 population doublings in culture through forced immortalization, showing that both perturbed states are stably propagated if not irreversible.

The observations made here, in turn, raise the question of cell-of-origin. We demonstrate through transcriptome-wide analysis, RT-qPCR, immunostaining and growth characteristics that the iCAF perturbed state mirrors the interlobular steady-state, whereas the myCAF state shares identity with the lobular steady state. Such unidirectional differentiation, rather than context-dependent differentiation or phenotypic convergence going from the steady to perturbed state, has been found through genetic lineage tracing in mice to be operating among reticular and papillary dermis fibroblast lineages [36–38]. The same conclusion was reached as CD26^+^ and CD26^−^ mammary fibroblasts transitioned to iCAFs and myCAFs, respectively [39], despite the obvious anatomic differences between mouse and human mammary tissue (reviewed in [40]). Moreover, in agreement with the present work, the absence of the iCAF signature is observed among CD26^−^ CAFs, whereas the single-cell RNA-Seq-based myCAF signature is more promiscuous and to some extent shared between CD26^−^ and CD26^+^ CAFs. However, in silico*-*based inference of origin using trajectory analysis suggests that both normal subtypes transition to iCAFs and then to myCAFs [39]. The clear discrepancy between the in silico trajectory analysis and the experimental evidence warrants caution in interpretation of purely in silico-based trajectory analyses. The present work not only offers an alternative explanation for the origin of iCAFs and myCAFs, it also brings about a cellular toolbox containing two major fibroblast lineages as present in the steady- and perturbed-state breast. To our knowledge, such a resource is not currently available from any other tissue.

Previous attempts to explain the heterogeneity among CAFs in mice have implicated recruitment of bMSCs which in general should facilitate metastatic potential [30–32]. Obviously, from a therapeutic point of view, such immigrated cells would be key to identify. Considering the similarities across tissues in universal steady states [41], it is difficult to deduce mesenchymal origin outside the breast based on RNA transcriptomic profiling. As an alternative, we subjected the myCAF cell line and crude primary CAFs to an ultimate experimental test probing the presence of bMSCs by osteogenic capacity in vivo. As it turned out, both immortalized and primary breast CAFs failed to form bone, indicating that even if present, the contribution of bMSCs to breast stroma is minimal. Our results align with the case report-based observations made in breast and other cancers in female patients after sex-mismatched bone marrow transplantation, which reported infrequent myofibroblasts harboring a Y chromosome [42]. Moreover, another recent study failed to identify bMSC recruitment to mammary tumors in mice [39].

We show here that while steady-state fibroblasts exhibit minimal contractility under the given culture conditions, both perturbed-state CAFs have acquired such properties. In contrast, we show that the CD105^high^ lineage, irrespective of steady or perturbed state, exerted tumor supportive function in vivo. The CD105^low^ lineage, again independent of state, was merely permissive for tumor growth. The experiments were carried out with ER^+^ breast cancer cells in combination with CAF cell lines from this particular breast cancer subtype. Whether or not other breast cancer subtypes are regulated in a similar manner cannot be extrapolated from the present data and should instead be addressed in future experiments. Nevertheless, the results challenge the view that CAFs, and not normal-derived fibroblasts, exert tumor supportive functions [23, 43]. This is relevant to keep in mind in a clinical setting if CAFs are pursued from the point of view of a normalization (reviewed in [34]). It is possible that some aspects of CAF function can be reverted, like contractile properties, but apparently the mechanisms behind the tumor support overrides those once in vivo. While the mechanism for tumor support warrants further investigation, the results suggest that in the human breast shared features are preserved across the steady and perturbed states.

## Conclusions

In the present work, establishment of CAF cell lines stably propagated in the iCAF and myCAF states and comparison with normal-derived counterparts suggests origin in steady-state interlobular and lobular fibroblasts, respectively. While the study does not exclude that other CAF subtypes may be relevant at different steps in tumor progression and for other breast cancer subtypes, the pro-tumorigenic support and clinical association with reduced breast cancer survival described here highlights the importance of fibroblasts of lobular origin not only in tumor initiation but also as tumors progress.

## Methods

### Tissue

Breast biopsies were collected from women undergoing mastectomy for primary breast cancer at the State University Hospital, Rigshospitalet. Donors were informed before surgery and agreed by written consent to donate tissue. The use of human material has been reviewed and approved by the Regional Scientific Ethical Committees (Region Hovedstaden, with reference to H-2-2011-052 and H-3-2010-095). No information about the donors is available to the authors. Material from some of the donated tissue has been included in previous studies. Eighteen tumors were collected and processed for histology and/or cell culture as described below. A total of twenty-seven human breast primary tumors, including eight of the above and nineteen from previously anonymously donated archival material, were included for analysis of CD105 expression by immunohistochemical staining. The biopsy material included in this work is anonymized.

### Cell isolation and cell culture

Upon excision, breast tumors were placed in serum-free DMEM-F12 (DMEM:Ham’s F12 Nutrient Mixture (F12), 1:1 v/v, Life Technologies), stored at 4 °C and collected within 24 h and processed as described in the following. Fourteen breast tumor biopsies were cut with scalpels to approximately 2mm^3^ and digested with ~ 5-mL collagenase solution (Worthington Biochemicals, 900 units/mL in serum free DMEM-F12) overnight at 37 °C on a rotary shaker at 60 RPM as previously described [8]. The digested material was rinsed twice in PBS solution and plated in Primaria™ T25 flasks (Beckton Dickinson) in DMEM-F-12 supplemented with 1% fetal bovine serum (FBS, Sigma), 2 mM glutamine (Gibco) and penicillin–streptomycin (PenStrep, Corning) antibiotics. The cultures were split at a ratio up to 1:3 onto collagen-coated flasks (Nunc, 8 μg collagen/cm^2^, PureColl, Cell Systems) from passage one onwards in DMEM-F-12 medium containing 5% FBS, PenStrep and glutamine (1% Glutagro or 2 mM glutamine, Gibco)) supplementation, referred to as DMEM-F12 5%, until direct use or for further analysis and sorting by FACS. After sorting by FACS, the cells were cultured under the same conditions with plating of 5600 cells/cm^2^ and passaged at every ~ 7–10 days.

Normal breast-derived steady-state fibroblast cell lines, iHBFC^CD105^ and iHBFC^CD26^ [5], here referred to as lobular and interlobular, respectively, were maintained in DMEM-F12 5% and passaged every ~ 7–10 days at 5600 cells/cm^2^ on collagen-coated tissue culture plastic (Nunc).

A bone marrow-derived mesenchymal stem cell (bMSC) line immortalized with hTERT [44] was cultured on plastic (Nunc) in Minimal Essential Medium (Gibco) supplemented with 10% FBS (South American Origin, Gibco) and 1% PenStrep. The bMSC line was maintained by splitting ~ 1:4 at 80% confluence.

An invasive subvariant of MCF7 breast cancer cells [45] was maintained in DMEM-F12 5% on regular non-coated plastic and split 1:20–1:5 at ~ 90% confluence.

Population doublings recorded from iCAFs and myCAFs were calculated as follows: *n* = 3.32(LogUCY–LogI) + X, where *n* = population doublings, UCY = cell yield, *I* = inoculation number and *X* = population doubling of inoculum. All cell cultures were incubated at 37 °C in a humidified atmosphere with 5% CO_2_.

### Tumor xenografts

Procedures for the tumor xenograft studies conducted at Lund University, Sweden, were approved by the ethical committee for animal experimentation under the Lund University ethical permit Dnr5.8.18–14,122/2020. Xenografts were established in highly immunocompromised mice (CIEA-NOG, Taconic, Denmark) by injecting 0.3 million MCF7 cancer cells mixed with or without 0.7 million fibroblasts. The cells were suspended in PBS + 10% FBS, and 50 μL cell suspension was orthotopically injected into the 4th left and 4th right mammary fat pad. Five groups were included: without fibroblasts (9 mice, 14 tumors), interlobular fibroblasts (4 mice, 8 tumors), lobular fibroblasts (4 mice, 8 tumors), iCAFs (4 mice, 7 tumors) and myCAFs (4 mice, 7 tumors).

Tumor volume was measured weekly using a caliper and calculated using the formula: *x*^2^*y*3.14/6, where x is the smallest diameter and y the largest diameter of the tumor. Tumors that reached ≥ 20 mm^3^ at endpoint were considered established. Mice received supplementation with beta-estradiol (E2758, Sigma) in the drinking water at 0.67 μg/mL from the day of injection throughout the duration of the experiment. The supplemented water was replenished biweekly.

### In vivo bone formation assay

Procedures for injection of human cells under the skin were reviewed and approved by the Danish National Animal Experiment Inspectorate (2017-15-0201-01210). One million bMSCs (7 implants, 3 mice), myCAFs (4 implants, 3 mice) and crude primary CAFs from three different donors in passage five (13 implants, 5 mice) were mixed with 40 mg hydroxyapatite/tricalcium phosphate (HA/TCP) ceramic powder (Zimmer Scandinavia, Albertslund, Denmark), incubated at 37 °C at 5% CO_2_ atmosphere overnight and then implanted subcutaneously in the dorsal side of immune-deficient mice (NOD.CB17-Prkdc^Scid^/J, Charles River, France). Implants were removed after eight weeks, transferred to 4% neutral buffered formalin for 24 h followed by incubation in formic acid for 3 days. The processed implants were paraffin embedded, sectioned and stained as described [46] with human-specific vimentin (clone SP20) antibody and hematoxylin ± eosin. The primary CAFs included in this experiment were cultured under conditions specified above for breast fibroblasts as well as those described for bMSCs, and the number of implants refers to the combined total. Mice transplanted with bMSCs served as a positive control for bone formation and material from one of these experiments was included in a previous study [5].

### Gel contraction assay

A collagen solution at final concentration (2.5 mg/mL) for contraction was prepared by adding 8:10 collagen (Purecol, Cell systems, 5005), 1:10 10X PBS with phenol red indicator adjusted to neutral pH with 0.1 M NaOH. 50.000 fibroblasts were suspended in 100 μL collagen solution and were plated in quadruplicate wells of 96-well plates pre-coated with 1% bovine serum albumin (1 h). After gelification at 37 °C, 100 μL DMEM-F12 5% medium per well was added and a scanning photograph acquired immediately after and at the indicated time points. Two experiments were conducted in technical quadruplicate. Analysis of gel area was performed with image analysis software ImageJ (version 1.53c), and results are expressed in gel area as percentage of initial gel on day 0 and statistical analysis used one-way ANOVA with Benjamini–Hochberg multiple test correction.

### Fluorescence-activated cell sorting (FACS)

CAF1 (passage 8), CAF2 (passage 13) and CAF3 (passage 9 and 14) were suspended to single cells through trypsinization and incubated with CD105-AF488 (SN6, 1:25) for 30 min followed by two washes in HEPES buffer. Fixable Viability Stain 780 (1:1000, BD Bioscience) was added prior to analysis and served as live dead discriminator. Unstained cells served as control. Analysis and sorting were performed on FACS ARIA II (BD Bioscience). CAFs were sorted into CD105^low^ (CAF^low^) and CD105^high^ (CAF^high^) populations, and the cells were further subcultured as described above. Freshly sorted CAF^low^ and CAF^high^ from CAF1 and CAF2 were smeared for staining with α-sm-actin following the methanol fixation-based protocol detailed above and staining with DAPI before evaluation and acquisition of images with an epi-fluorescence microscope (Leica DM5500B). A minimum of 100 cells were evaluated for α-sm-actin, categorized as positive or negative and expressed as percent of total number of cells evaluated.

### Immunohistochemistry and immunocytochemistry

Immunoperoxidase-based stainings of cryostat sections (6–8 um) and cell cultures were conducted essentially as previously described [47–49]. Briefly, cryostat sections and cell cultures underwent fixation at – 20 °C in 100% methanol (Fixation M in Table 2) or in Formaldehyde (Fixation F in Table 2) followed by permeabilization by 0.1% TritonX-100 with (F1 in Table 2) or without (F2 in Table 2) intermediate incubation with methanol:acetone (1:1, *v:v*). For blocking, the sections were incubated with Ultra V blocking solution (Lab Vision Corporation, TA125-UB) or 10% Goat serum in PBS for 5–10 min at room temperature (RT) followed by 60-min incubation with primary antibody. The sections were rinsed twice in PBS for 10-min incubation before adding secondary antibody UltraVision ONE HRP polymer (Thermo Fisher, TL-125-PHJ) for 30 min at RT. For stain development, sections were rinsed in PBS and then incubated with 3,3’-Diaminobenzidine for 10 min at RT. Antibodies used are listed in Table 2.

**Table 2:** List of antibodies and protocols

Antibody	Clone/isotype	Company/Catalogue no	Peroxidase	Immuno-fluorescence	FACS	Fixation
CD105-AF488	SN6/IgG1	AbD Serotec/MCA1557A488			1:25	
CD105	SN6	Abcam/Ab11414	1:100			M
CD26	202–36	Abcam/Ab3154	1:50			M
CD140b	PR7212	R&D Systems/MAB1263	1:200			F1
Tenascin	BC-24	Sigma, T2551	1:5000			M
Vimentin	SP20	ThermoFisher Scientific/RM-9120	1:200			FFPE
α-sm actin	1A4	Sigma/A2547	1:500–1:1000	1:1000		M
K19	A53b/a2	Abcam/ab7754		1:1000		M
CD31	89C2	Cell Signaling/3528S		1:1000		M
MYH11	SMMS-1	DAKO/M3558	1:50			M
CD146/MCAM	P1H12	Abcam/Ab24577	1:500			M
GATA3	HG3-31	Santa Cruz/sc-268	1:250			F2
ER	EP1	Dako/M3643	1:50			F2
PR	PgR636	Dako/M3569	1:100			F2
K5	XM26	Novocastra/NCL-L-CK5	1:250			M
K14	LL002	Monosan/MonX10687	1:100			M
HER2	TAB250	Invitrogen/28-0003Z	1:200			M
AF594	Anti-mouse	Invitrogen/A21203		1:500–1:1000		
AF568	Anti-mouse IgG2a	Invitrogen/A21134		1:500		

FACS sorted smeared cells were fixed in ice-cold methanol at – 20 °C for 5 min, left to dry before commencing with blocking (Ultra V blocking solution) for 5 min and incubation with anti-α-sm actin (1A4, Sigma) primary antibody for 60 min, interrupted by washing in PBS, before incubation with a fluorophore-labeled secondary antibody.

### Viral transduction

Constructs used were: human telomerase (pBabe-neo-hTERT, Addgene #1774, a gift from Robert Weinberg [50], empty vector (pBabe-neo, addgene # 1767, a gift from Hartmut Land & Jay Morgenstern & Robert Weinberg [51], and viral packaging construct pCL-Ampho (a gift from Hung Nguyen [52].

Retroviral particles ± the hTERT construct were generated as described previously [5]. CAFs from three biopsies in passage four at 90% confluency were transduced with the viral particles at serial dilution overnight and then rinsed. In passage five, at 80% confluency, the transduced cells underwent antibiotic selection with medium containing 300 μg/mL G418 (Life Technologies) for two weeks until live non-transduced cells were eliminated. The transduction efficiency was less than 15%, hence most cells were transduced by one copy of retroviral particle.

### RNA extraction, RT-qPCR, RNA sequencing and bioinformatics

Total RNA was isolated according to manufacturers’ instructions using Trizol (Thermo Fisher) and a spin column method (Zymo Research) from subconfluent cell cultures in duplicate of CAF1-derived CAF^low^ (iCAFs, passage 33) and CAF^high^ (myCAFs, passage 32) and in triplicate from CAF2-derived CAF^low^ (passage 18) and CAF^high^ (passage 18) and in duplicate from CAF3-derived CAF^low^ (passage 9) and CAF^high^ (passage 9). RNA from interlobular fibroblasts (passage 25) and lobular fibroblasts (passage 24) each in duplicate subconfluent cell culture was previously extracted as above and analyzed by RNA-Seq [5].

For reverse transcription quantitative polymerase chain reaction (RT-qPCR) the total RNA was reverse transcribed to cDNA using the High-Capacity RNA-to-cDNA Kit (Applied biosystems). Taq-Man gene expression assays (Applied biosystems) were used for RT-qPCR listed in Table 3. The delta (Δ)ΔC(t) method given by the formula 2^−ΔΔC(t)^ [53] was used for analyzing gene expression including the use of the geometric mean of four reference genes (Table 3). Each sample replicate was analyzed in technical duplicate per gene. In each sample set, interlobular fibroblasts and CAF^low^ were set as reference for calculating fold changes of gene expression relative to lobular fibroblasts and CAF^high^, respectively. For plotting in heatmap, fold changes were log10-transformed. Unpaired Student’s t test was used to calculate *p*-values on gene expression fold changes.

**Table 3:** List of Taq-Man gene expression assays for RT-qPCR

Assay ID	Gene symbol	Gene name	Marker
Hs00163811_m1	*C3*	Complement C3	iCAF
Hs00998018_m1	*PDGFRA*	Platelet Derived Growth Factor Receptor Alpha	iCAF
Hs00236937_m1	*CXCL1*	C-X-C Motif Chemokine Ligand 1	iCAF
Hs00174103_m1	*CXCL8*	C-X-C Motif Chemokine Ligand 8	iCAF
Hs01555410_m1	*IL1B*	Interleukin 1 Beta	iCAF
Hs00605742_g1	*CXCL6*	C-X-C Motif Chemokine Ligand 6	iCAF
Hs00983056_m1	*CDH2*	Cadherin 2	Interlobular
Hs00247429_m1	*DKK3*	Dickkopf WNT Signaling Pathway Inhibitor 3	Interlobular
Hs01675818_s1	*TWIST1*	Twist Family BHLH Transcription Factor 1	Interlobular
Hs00392834_m1	*SULF1*	Sulfatase 1	Interlobular
Hs00183105_m1	*LPXN*	Leupaxin	Interlobular
Hs01027737_m1	*MFAP2*	Microfibril Associated Protein 2	Interlobular
Hs00923996_m1	*ENG*	Endoglin (CD105)	Lobular
Hs04400911_m1	*MFAP5*	Microfibril Associated Protein 5	Lobular
Hs00738371_m1	*SCUBE3*	Signal Peptide, CUB Domain And EGF Like Domain Containing 3	Lobular
Hs00535586_s1	*CD248*	CD248 Molecule	Lobular
Hs00740811_m1	*FHL1*	Four And A Half LIM Domains 1	Lobular
Hs00162844_m1	*CLEC3B*	C-Type Lectin Domain Family 3 Member B	Lobular
Hs00909449_m1	*ACTA2*	Actin Alpha 2, Smooth Muscle	myCAF
Hs00921987_m1	*CALD1*	Caldesmon 1	myCAF
Hs00154543_m1	*CNN1*	Calponin 1	myCAF
Hs00365052_m1	*FN1*	Fibronectin 1	myCAF
Hs00162558_m1	*TAGLN*	Transgelin	myCAF
Hs00235006_m1	*ITGA1*	Integrin Alpha 1	myCAF
Hs01060665_g1	*ACTB*	Actin Beta	Reference
Hs02758991_g1	*GAPDH*	Glyceraldehyde-3-Phosphate Dehydrogenase	Reference
Hs00951083_m1	*TFRC*	Transferrin Receptor	Reference
Hs00943178_g1	*PGK1*	Phosphoglycerate Kinase 1	Reference

For RNA-Seq, the RNA extracted from subconfluent duplicate cultures of CAF1-derived CAF^low^ (iCAFs) and CAF^high^ (myCAFs) was used. RNA sequencing and sequence alignment was performed at the Beijing Genomics Institute (BGI), Hong Kong. Sequencing was performed as previously described [5]. Briefly, a DNBseq platform was used for sequencing and generating on average ~ 24 million clean reads per sample. The clean reads were mapped to the reference genome using HISAT2 [54]. Clean reads were mapped to reference transcripts using Bowtie2 [55]. This iCAF/myCAF dataset was integrated with a RNA-Seq dataset from lobular and interlobular fibroblasts [5]. Differentially expressed genes were identified using the DESeq2 method [56] including statistical testing using Wald test corrected for multiple testing using the Benjamini–Hochberg method to calculate adjusted *p*-values. The average FPKM transcript level of the technical duplicates in the iCAF/myCAF and lobular/interlobular datasets was used to calculate gene expression fold changes between samples. Different FPKM cutoff levels were applied to different signatures as indicated in each analysis. A lower threshold of five FPKM was considered expressed, whereas a higher threshold of 50–100 FPKM was considered abundantly expressed. The genes included in each signature were statistically significant by the DESeq2 method with maximal adjusted *p*-value < 0.05. The FPKM method was used here for between samples comparison in agreement with the recommendations by Evans et al. [57] and Zhao et al. [58]: same mRNA extraction protocol was used, similar total RNA per cell per sample, similar low ribosomal RNA fraction (~ 1%) per sample, similar gene expression in total FPKM per sample, similar gene distribution per sample and similar per sample geometric mean of the top ranked reference genes (*IK, KDELR1, LAPTM4A, EEF2, SF3B2*) for human breast RNA-Seq data identified by the Housekeeping and Reference Transcript Atlas (https://housekeeping.unicamp.br/) [59]. Gene signatures for all analyses are provided in Additional file 9.

We used GSEA software (version 4.1.0) to perform gene set enrichment analysis (GSEA). Interlobular and lobular gene sets were defined a priori by extracting the differentially expressed genes (≥ 5 FPKM, ≥ 2-fold change, adjusted *p* < 0.05) between the two cell types, resulting in 409 and 482 genes, respectively. Each gene in the full iCAF/myCAF dataset containing gene expression for 17,689 genes was ranked using the signal-to-noise ranking metric in the GSEA software. GSEA software was used to determine whether the interlobular and lobular gene sets exhibited enrichment in the ranked iCAF/myCAF dataset. Data are presented as gene set enrichment plots with statistics.

KEGG and Reactome pathway enrichment tests were performed using the compareCluster() function (pvalueCutoff = 0.05) of the clusterProfiler package (version 4.2.2, [60]) on DEGs (iCAF = 295 genes, myCAF = 413 genes) from the DESeq2-analysis filtered for log_2_FC > 4.0 or log_2_FC < -4.0 and adjusted *p* < 0.001 between myCAFs and iCAFs. All genes from the dataset were used as background for the enrichment analysis.

STRING.db software (https://string-db.org/, version 11.5) was used for pathway enrichment analysis on smaller differentially expressed gene lists (≥ 50 FPKM, ≥ 2-fold change, adjusted *p* < 0.001) comparing iCAFs and myCAFs using the Whole Genome setting for statistical background.

Breast cancer TCGA expression data [61–63] and corresponding clinical information, i.e. disease-specific survival data, was downloaded using the cgdsr package (version 1.3.0) provided by the cBioportal database [64, 65]. For survival analysis we defined iCAF and myCAF gene signatures as the genes differentially expressed (≥ 100 FPKM, ≥ 2-fold change, adjusted *p* < 0.0001) between iCAFs and myCAFs. TCGA data on patients with 10-years follow-up and 15-years follow-up were split into iCAF and myCAF signature expression tertiles. Patient survival analysis was performed using the Surv() and survfit() functions in the survival package (version 3.5–5, [66]). The Kaplan–Meier plots were drawn using the ggsurvplot() function in survminer (version 0.4.9, [67]). The log-rank test was used for statistical assessment of the survival time analyses. The PAM50 (Prediction Analysis of Microarrays 50 gene signature, [68]) molecular subtype (Luminal A, Luminal B, Basal, Her2, and Normal-like) calls were obtained from the downloaded TCGA breast cancer clinical data file.

The breast cancer single-cell RNA-seq dataset [17] was downloaded from Broad Institute’s Single Cell Portal (https://singlecell.broadinstitute.org/single_cell). The dataset was then processed using the basic pipeline of Seurat (version 4.3.0, [69]). Cell-type annotations and UMAP coordinates were provided by the authors. Seurat’s AddModuleScore() function was used to calculate expression levels of iCAF and myCAF gene signatures using the top 65 (according to fold-change) differentially expressed genes (≥ 100 FPKM, ≥ 2-fold change, adjusted *p* < 0.0001) between iCAFs and myCAFs. Clusters and gene signatures were visualized using the scCustomize package (version 0.7.0, [70]) on a 2D map produced with the UMAP method.

A CAF marker signature was derived by collecting the genes and individual genes of gene families that were defined as positive CAF markers in three reviews [26, 28, 29]. The expression of these 38 genes was extracted from the RNA-Seq datasets for iCAFs, myCAFs, interlobular and lobular fibroblasts and the 31 genes that were expressed above 5FPKM plotted in a heatmap for visualization. Analysis of enrichment of CAF markers was conducted by counting the genes (15) expressed twofold higher in either CAF versus both interlobular and lobular fibroblasts. None of the genes were expressed twofold higher in either steady-state fibroblast versus both iCAFs and myCAFs. Enrichment was tested by Fisher’s exact test using the total number of genes analyzed, 17,689, as reference list.

### Statistics

Statistical analysis and data visualization were conducted with Microsoft Excel, statistical programming software R and Rstudio (version 2022.07.01 build 554) and GSEA (version 4.1.0, [71]). Statistical tests were unpaired Student’s test, one-way ANOVA with Benjamini–Hochberg multiple test correction, Fisher’s exact test and log-rank test.

### Supplementary Information

**Additional file 1**. **Figure S1a** and **b**: Identification of CD105^low^ and CD105^high^ CAFs in culture and in situ. a) FACS plots of CAF2 (left, passage 13) and CAF3 (right, passage 14) single-cell suspended and labeled by immunofluorescence with a CD105 antibody (x-axis) versus forward scatter (y-axis). Circles indicate CD105^low^ (CAF^low^) and CD105^high^ (CAF^high^) cells. b) Bar plot of quantification of percentage of α-sm actin positive cells in smears from FACS sorted CAF1 and CAF2 into CAF^low^ (gray bar) and CAF^high^ (black bar). Note the relatively few α-sm actin positive cells among the CAF^low^ population in both CAF1 and CAF2. Quantification in CAF3 was not done.

**Additional file 2**. **Figure S1c**: Identification of CD105^low^ and CD105^high^ CAFs in culture and in situ. c) Micrographs of representative regions within a single cryostat section of the primary tumors CAF1, CAF2 and CAF3 immunoperoxidase-stained for CD105 and counterstained by hematoxylin (nuclei). In all three tumors, cancer cells (arrowheads) are surrounded by both CAF^low^ (left, arrows) and CAF^high^ (right, arrows). Scale bar = 50 μm.

**Additional file 3**. **Table S1**: Pathways enriched in iCAFs and myCAFs. Selected pathways enriched in iCAFs (upper) and myCAFs (lower, ≥50 FPKM, ≥2-fold change, adjusted p<0.001) obtained from STRING network analysis (https://string-db.org/, version 11.5). All pathways are provided in Additional file 10.

**Additional file 4.** **Figure S2**: CAF^low^ and CAF^high^ represent iCAFs and myCAFs, respectively. a) Heatmap depicting the expression level fold change of six iCAF and six myCAF genes measured by RT-qPCR in CAF^high^ expressed relative to CAF^low^ derived from CAF1 (left), CAF2 (middle) and CAF3 (right). Color key represents the log10-transformed CAF^high^/CAF^low^ fold change. Numbers are the p-values by Student’s unpaired t-test. b) Micrographs of CAF^low^ (upper) and CAF^high^ (lower) derived from CAF1 (left), CAF2 (middle) and CAF3 (right) immunoperoxidase-stained against α-sm actin (brown) and counterstained by hematoxylin for nuclei (blue). Irrespective of origin, CAF^high^ exhibit prominent positive staining which is relatively infrequent in CAF^low^. Scale bar = 100 μm.

**Additional file 5**. **Figure S3**: CAF^low^ are interlobular-like and CAF^high^ are lobular-like. a) Micrographs of CAF^low^ and CAF^high^ derived from CAF2 and CAF3 immunoperoxidase-stained against CD105, tenascin and CD26. Note that irrespective of origin, the staining profiles with respect to CD105 and tenascin in CAF^low^ and CAF^high^ correspond to those of interlobular fibroblasts and lobular fibroblasts, respectively (for comparison see Fig. 1d and 3a). The absence of CD26 in both CAFs indicates that this marker of the interlobular fibroblast lineage is state-dependent. Scale bar = 100 μm. b) Heatmap depicting gene expression fold changes determined by RT-qPCR in lobular fibroblasts relative to interlobular fibroblasts, myCAFs relative to iCAFs and in CAF^high^ relative to CAF^low^ derived from CAF2- and CAF3. Color key represents the log10-transformed fold changes. Numbers are p-values by Student’s unpaired t-test.

**Additional file 6**. **Figure S4a**: CAFs lack osteogenic differentiation capacity in culture and in vivo. a) Bar diagram of quantification of osteogenic differentiation among iCAFs, myCAFs and bMSCs upon exposure to control medium (-) or osteogenic inducing medium (OIM, +) determined after staining with alizarin red for which representative micrographs are shown. For myCAFs and bMSCs error bars represent +/- SD of three biological repeats each in technical duplicate. For iCAFs the bars represent the mean +/- SD of technical duplicates. One-way ANOVA with Benjamini-Hochberg multiple test correction comparing myCAFs and bMSCs found significant matrix mineralization among bMSCs between OIM and CM and between myCAFs OIM and bMSCs OIM (asterisk indicates p<0.001). ARBU: arbitrary units.

**Additional file 7**. **Figure S4b and c**: CAFs lack osteogenic differentiation capacity in culture and in vivo. b) Representative micrographs of sections from the xenograft in vivo bone formation assay immunoperoxidase-stained with a human-specific vimentin antibody for identification of the implanted cells and additional cellular staining with hematoxylin and eosin (H&E). Bone formation is absent in implants with crude, low passage primary CAFs (left), but forms upon grafting of bMSCs (right, arrow). Bar = 50 μm. c) Representative micrographs of sections from in vivo bone formation assay stained for human-specific vimentin and hematoxylin (upper) and H&E (lower), showing absence of bone formation by myCAFs (left panel), otherwise readily formed by bMSCs (right panel, white arrows). Bar = 50 μm.

**Additional file 8**. **Figure S5**: The myCAF signature is not associated with survival in a particular PAM50 breast cancer subtype. Kaplan-Meier curve showing breast cancer survival of PAM50 subtypes stratified according to low (<33%, blue) and high (>67%, red) expression of the myCAF signature (≥﻿100 FPKM, ≥2-fold change between myCAF versus iCAF, adjusted p< 0.0001) with 10- and 15 years follow-up in data from TCGA. Association was tested by the log-rank test and none reached the statistical significance level of 0.05. Number of patients at risk at the indicated time points are shown below each plot.

**Additional file 9**. Gene signatures for analysis of iCAFs, myCAFs, interlobular fibroblasts and lobular fibroblasts. Gene signatures applied for analysis of differentiation and association with breast cancer specific survival.

**Additional file 10**. All pathways enriched in iCAFs and myCAFs. All pathways enriched in iCAFs and myCAFs (≥50 FPKM, ≥2-fold change, adjusted p<0.001) obtained from STRING network analysis (https://string-db.org/, version 11.5).

## Data Availability

The RNA-Seq dataset on iCAFs and myCAFs generated and analyzed during the current study is available in the Gene Expression Omnibus (GEO) repository], [GEO accession number GSE244354, https://www.ncbi.nlm.nih.gov/geo/query/acc.cgi?acc=GSE244354) which includes the integrated dataset on interlobular and lobular fibroblasts previously deposited to the GEO repository (GSE153646, https://www.ncbi.nlm.nih.gov/geo/query/acc.cgi?acc=GSE153646).

## Abbreviations

α-sm actin: α-Smooth muscle actin
bMSC: Bone marrow-derived mesenchymal stem cell
CAF: Cancer-associated fibroblast
CD105: Cluster of differentiation 105
CD26: Cluster of differentiation 26
FACS: Fluorescence-activated cell sorting
FPKM: Fragments per kilobase of transcript per million mapped reads
hTERT: Human telomerase reverse transcriptase
iCAF: Inflammatory cancer-associated fibroblast
myCAF: Myofibroblast cancer-associated fibroblast
RNA-Seq: RNA sequencing
TCGA: The Cancer Genome Atlas database
TGF-β: Transforming growth factor-beta
UMAP: Unifold Manifold Approximation and Projection
