# Supplementary figures and images for: Evidence of steady-state fibroblast subtypes in the normal human breast as cells-of-origin for perturbed-state fibroblasts in breast cancer
Source: Breast Cancer Res. 2024 Jan 16;26:11. doi: 10.1186/s13058-024-01763-3 (PMC10790388; doi:10.1186/s13058-024-01763-3)

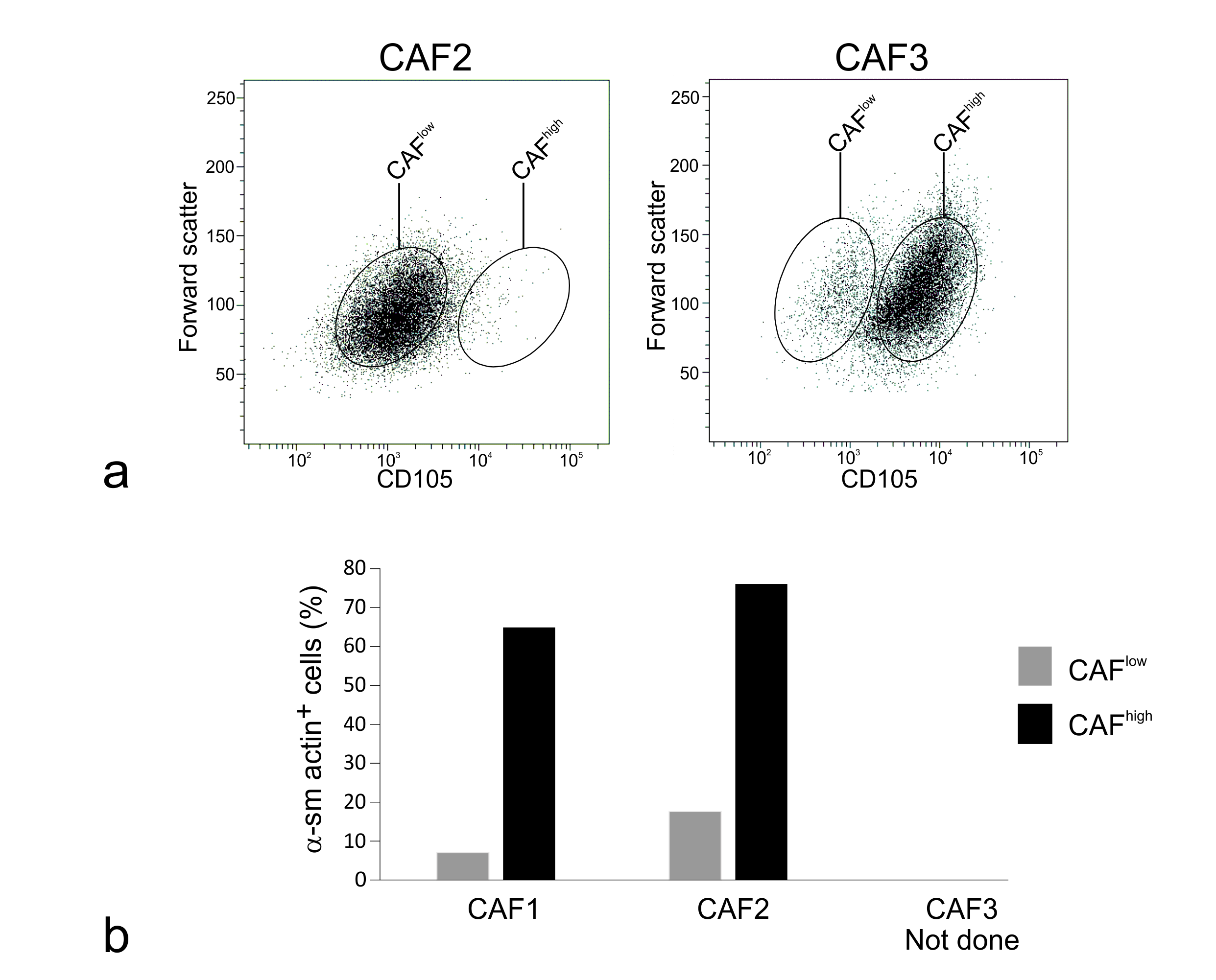

Supplement: Supplementary file 1 — Additional file 1. Figure S1a and b: Identification of CD105low and CD105high CAFs in culture and in situ. a) FACS plots of CAF2 (left, passage 13) and CAF3 (right, passage 14) single-cell suspended and labeled by immunofluorescence with a CD105 antibody (x-axis) versus forward scatter (y-axis). Circles indicate CD105low (CAFlow) and CD105high (CAFhigh) cells. b) Bar plot of quantification of percentage of α-sm actin positive cells in smears from FACS sorted CAF1 and CAF2 into CAFlow (gray bar) and CAFhigh (black bar). Note the relatively few α-sm actin positive cells among the CAFlow population in both CAF1 and CAF2. Quantification in CAF3 was not done. [file 13058_2024_1763_MOESM1_ESM.tif]

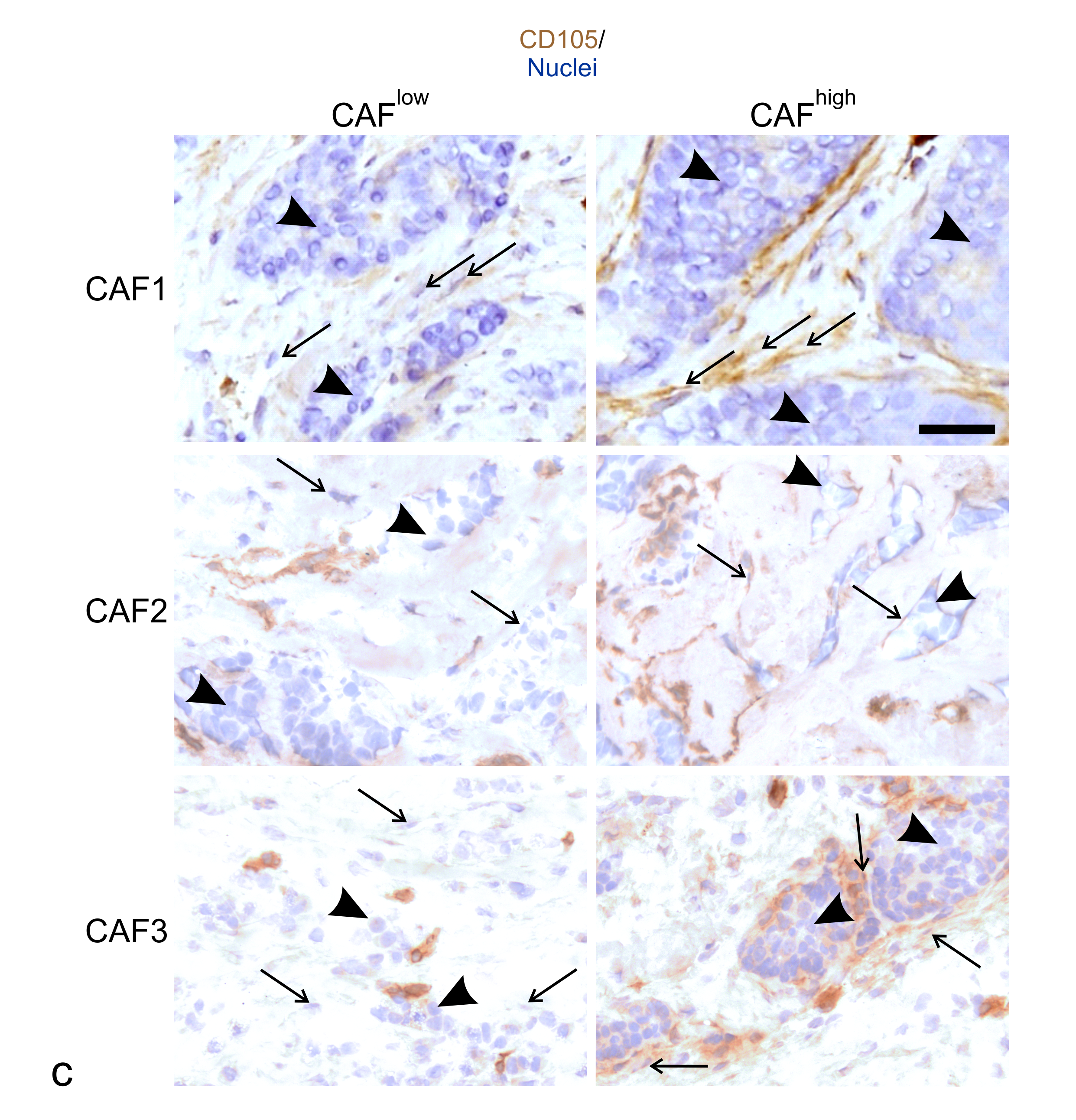

Supplement: Supplementary file 2 — Additional file 2. Figure S1c: Identification of CD105low and CD105high CAFs in culture and in situ. c) Micrographs of representative regions within a single cryostat section of the primary tumors CAF1, CAF2 and CAF3 immunoperoxidase-stained for CD105 and counterstained by hematoxylin (nuclei). In all three tumors, cancer cells (arrowheads) are surrounded by both CAFlow (left, arrows) and CAFhigh (right, arrows). Scale bar = 50 μm. [file 13058_2024_1763_MOESM2_ESM.tif]

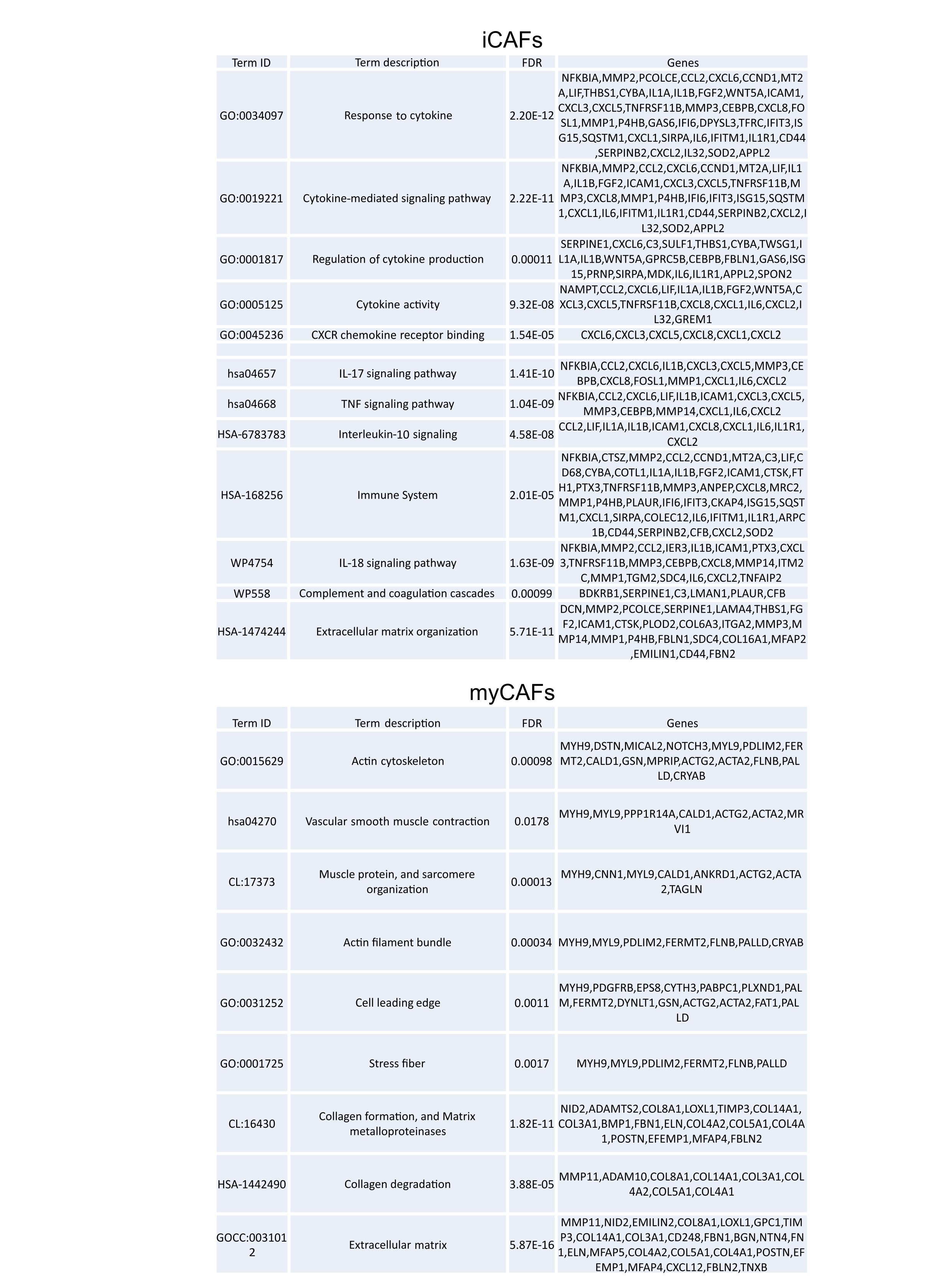

Supplement: Supplementary file 3 — Additional file 3. Table S1: Pathways enriched in iCAFs and myCAFs. Selected pathways enriched in iCAFs (upper) and myCAFs (lower, ≥50 FPKM, ≥2-fold change, adjusted p<0.001) obtained from STRING network analysis (https://string-db.org/, version 11.5). All pathways are provided in Additional file 10. [file 13058_2024_1763_MOESM3_ESM.tif]

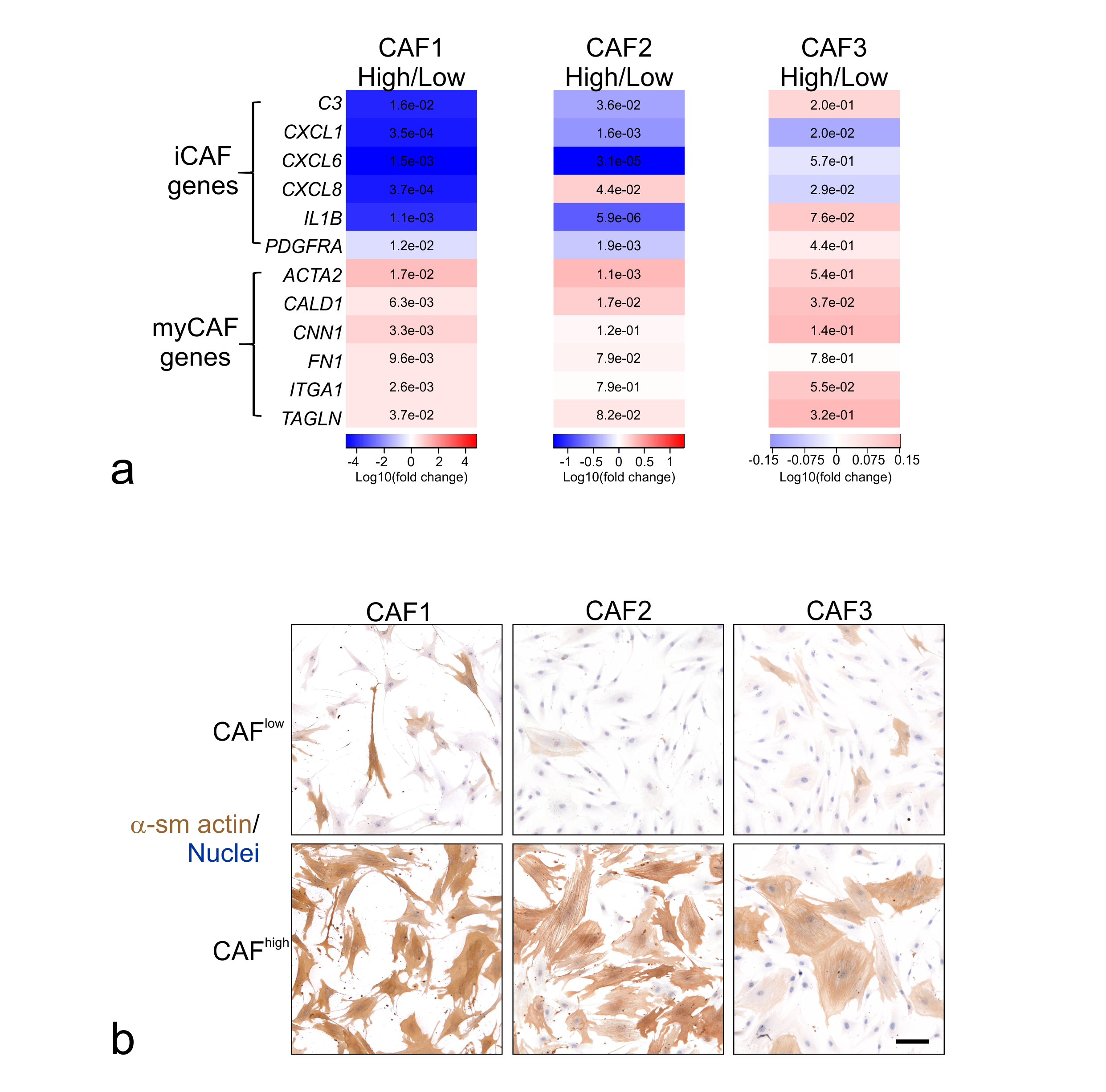

Supplement: Supplementary file 4 — Additional file 4. Figure S2: CAFlow and CAFhigh represent iCAFs and myCAFs, respectively. a) Heatmap depicting the expression level fold change of six iCAF and six myCAF genes measured by RT-qPCR in CAFhigh expressed relative to CAFlow derived from CAF1 (left), CAF2 (middle) and CAF3 (right). Color key represents the log10-transformed CAFhigh/CAFlow fold change. Numbers are the p-values by Student’s unpaired t-test. b) Micrographs of CAFlow (upper) and CAFhigh (lower) derived from CAF1 (left), CAF2 (middle) and CAF3 (right) immunoperoxidase-stained against α-sm actin (brown) and counterstained by hematoxylin for nuclei (blue). Irrespective of origin, CAFhigh exhibit prominent positive staining which is relatively infrequent in CAFlow. Scale bar = 100 μm. [file 13058_2024_1763_MOESM4_ESM.tif]

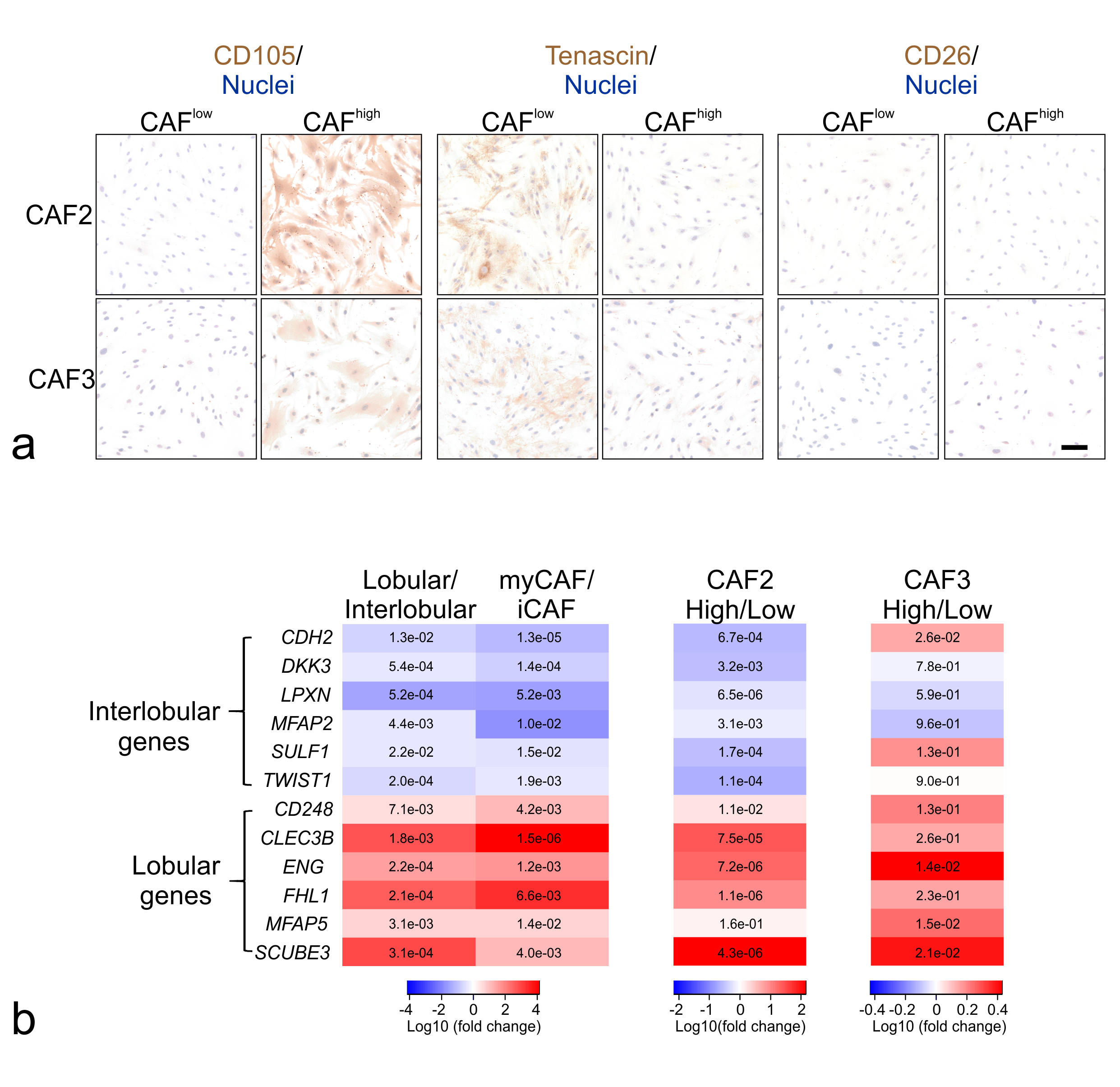

Supplement: Supplementary file 5 — Additional file 5. Figure S3: CAFlow are interlobular-like and CAFhigh are lobular-like. a) Micrographs of CAFlow and CAFhigh derived from CAF2 and CAF3 immunoperoxidase-stained against CD105, tenascin and CD26. Note that irrespective of origin, the staining profiles with respect to CD105 and tenascin in CAFlow and CAFhigh correspond to those of interlobular fibroblasts and lobular fibroblasts, respectively (for comparison see Fig. 1d and 3a). The absence of CD26 in both CAFs indicates that this marker of the interlobular fibroblast lineage is state-dependent. Scale bar = 100 μm. b) Heatmap depicting gene expression fold changes determined by RT-qPCR in lobular fibroblasts relative to interlobular fibroblasts, myCAFs relative to iCAFs and in CAFhigh relative to CAFlow derived from CAF2- and CAF3. Color key represents the log10-transformed fold changes. Numbers are p-values by Student’s unpaired t-test. [file 13058_2024_1763_MOESM5_ESM.tif]

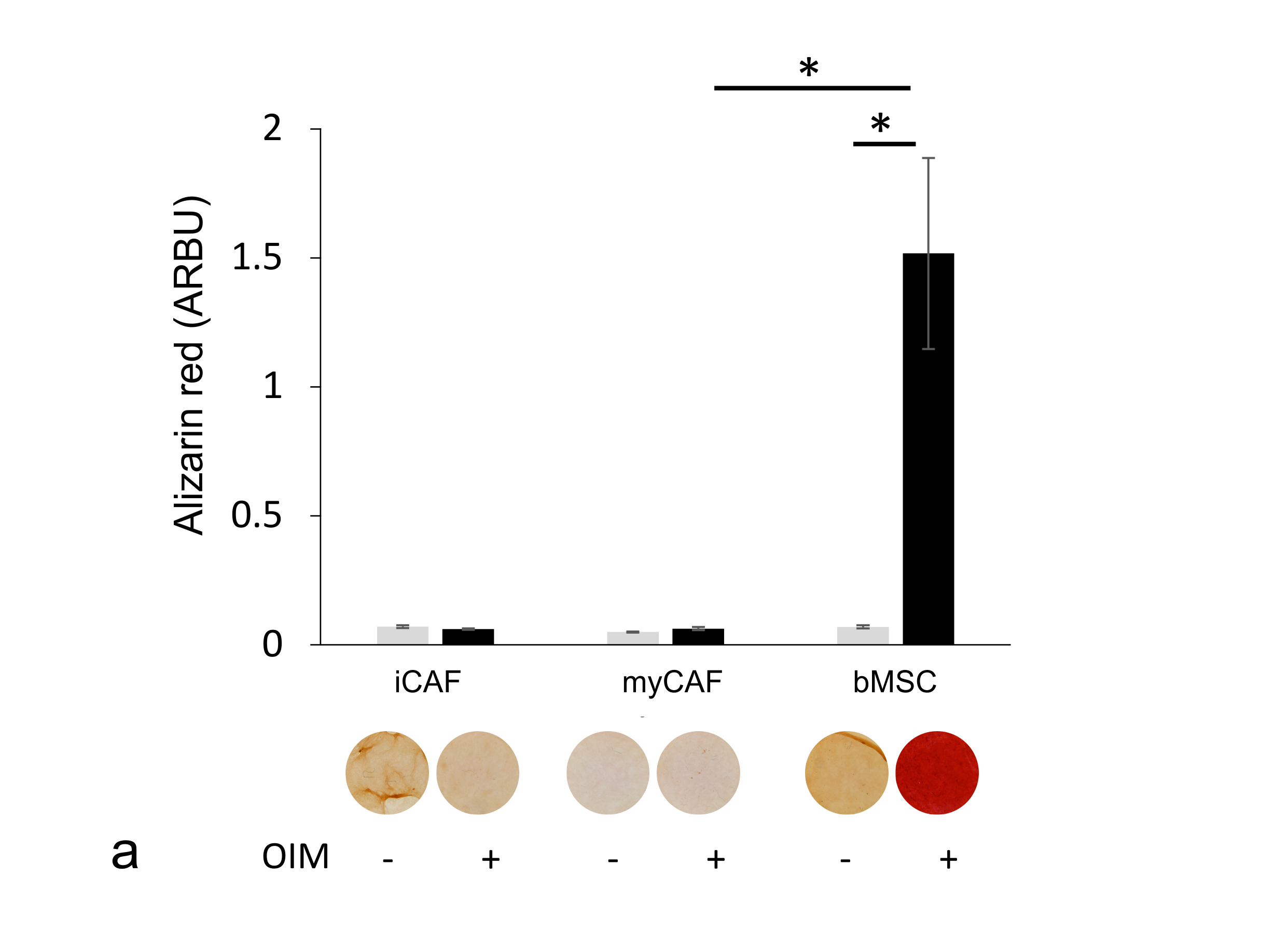

Supplement: Supplementary file 6 — Additional file 6. Figure S4a: CAFs lack osteogenic differentiation capacity in culture and in vivo. a) Bar diagram of quantification of osteogenic differentiation among iCAFs, myCAFs and bMSCs upon exposure to control medium (-) or osteogenic inducing medium (OIM, +) determined after staining with alizarin red for which representative micrographs are shown. For myCAFs and bMSCs error bars represent +/- SD of three biological repeats each in technical duplicate. For iCAFs the bars represent the mean +/- SD of technical duplicates. One-way ANOVA with Benjamini-Hochberg multiple test correction comparing myCAFs and bMSCs found significant matrix mineralization among bMSCs between OIM and CM and between myCAFs OIM and bMSCs OIM (asterisk indicates p<0.001). ARBU: arbitrary units. [file 13058_2024_1763_MOESM6_ESM.tif]

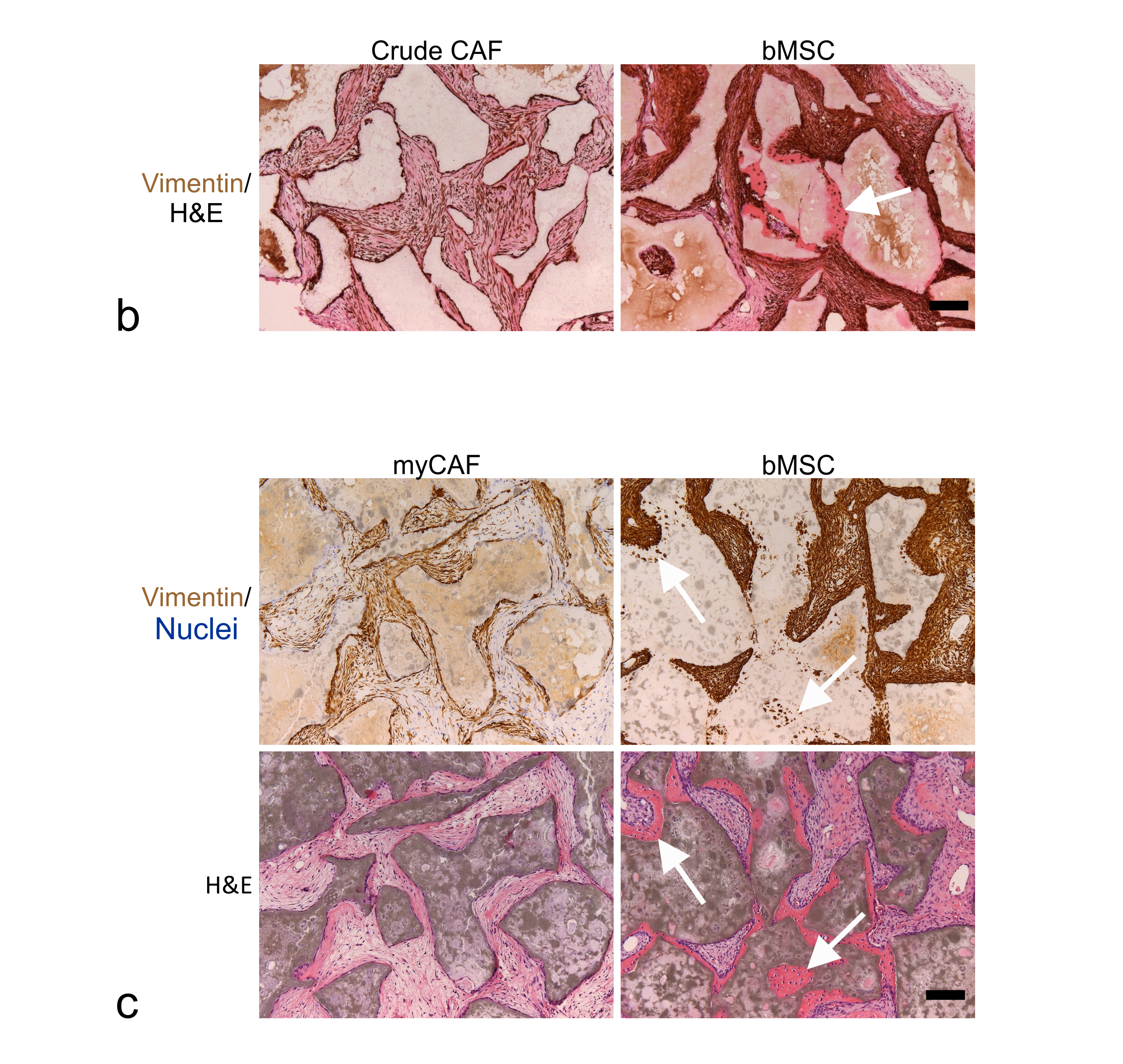

Supplement: Supplementary file 7 — Additional file 7. Figure S4b and c: CAFs lack osteogenic differentiation capacity in culture and in vivo. b) Representative micrographs of sections from the xenograft in vivo bone formation assay immunoperoxidase-stained with a human-specific vimentin antibody for identification of the implanted cells and additional cellular staining with hematoxylin and eosin (H&E). Bone formation is absent in implants with crude, low passage primary CAFs (left), but forms upon grafting of bMSCs (right, arrow). Bar = 50 μm. c) Representative micrographs of sections from in vivo bone formation assay stained for human-specific vimentin and hematoxylin (upper) and H&E (lower), showing absence of bone formation by myCAFs (left panel), otherwise readily formed by bMSCs (right panel, white arrows). Bar = 50 μm. [file 13058_2024_1763_MOESM7_ESM.tif]

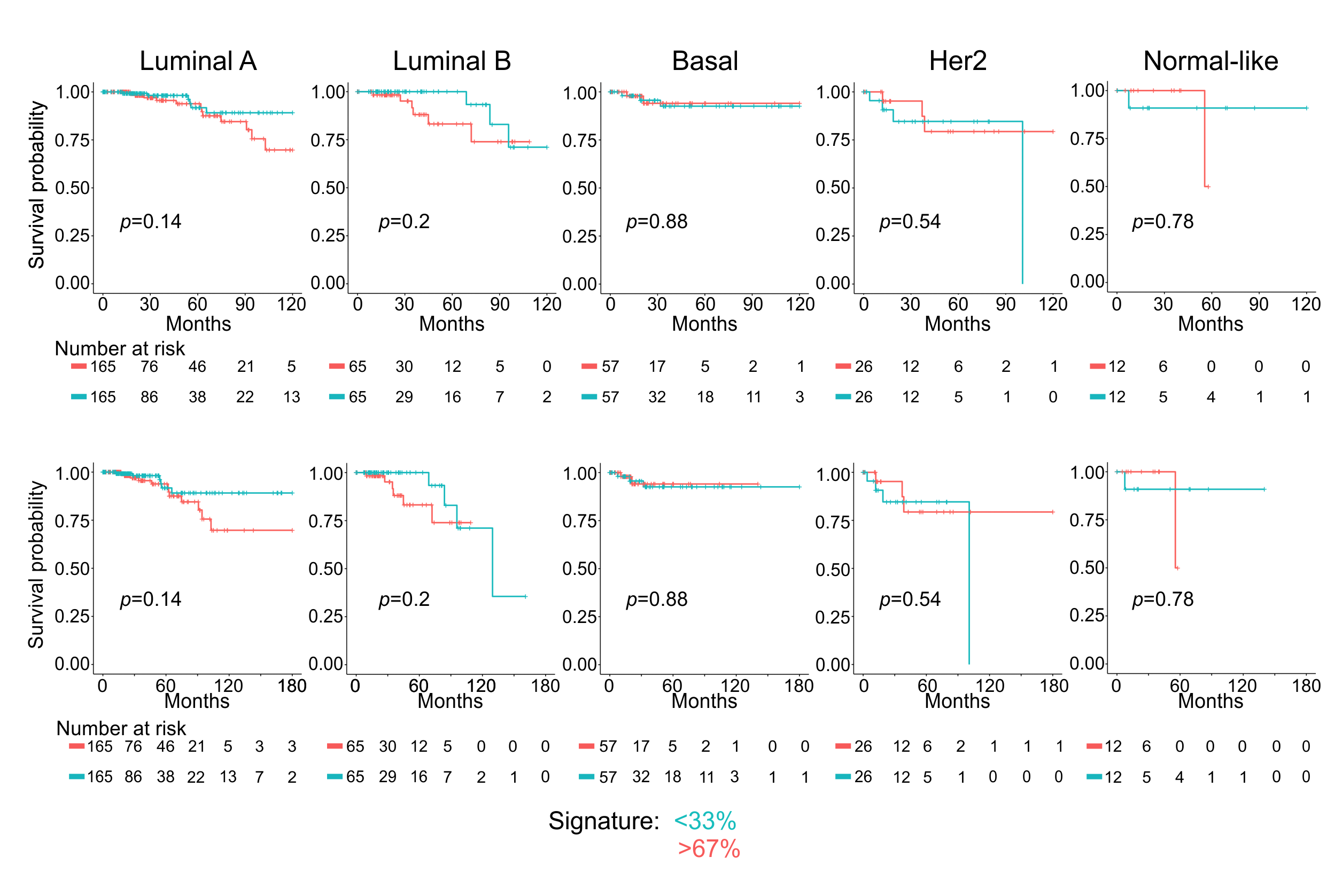

Supplement: Supplementary file 8 — Additional file 8. Figure S5: The myCAF signature is not associated with survival in a particular PAM50 breast cancer subtype. Kaplan-Meier curve showing breast cancer survival of PAM50 subtypes stratified according to low (<33%, blue) and high (>67%, red) expression of the myCAF signature (≥﻿100 FPKM, ≥2-fold change between myCAF versus iCAF, adjusted p< 0.0001) with 10- and 15 years follow-up in data from TCGA. Association was tested by the log-rank test and none reached the statistical significance level of 0.05. Number of patients at risk at the indicated time points are shown below each plot. [file 13058_2024_1763_MOESM8_ESM.tif]
